# Crosstalk between transcription factors and microRNAs in human protein interaction network

**DOI:** 10.1186/1752-0509-6-18

**Published:** 2012-03-13

**Authors:** Chen-Ching Lin, Ya-Jen Chen, Cho-Yi Chen, Yen-Jen Oyang, Hsueh-Fen Juan, Hsuan-Cheng Huang

**Affiliations:** 1 Graduate Institute of Biomedical Electronics and Bioinformatics, National Taiwan University, Taipei 106, Taiwan; 2 Genome and Systems Biology Degree Program, National Taiwan University, Taipei 106, Taiwan; 3 Institute of Biological Chemistry, Academia Sinica, Taipei 115, Taiwan; 4 Department of Life Science and Institute of Molecular and Cellular Biology, National Taiwan University, Taipei 106, Taiwan; 5 Institute of Biomedical Informatics, Center for Systems and Synthetic Biology, National Yang-Ming University, Taipei 112, Taiwan

## Abstract

**Background:**

Gene regulatory networks control the global gene expression and the dynamics of protein output in living cells. In multicellular organisms, transcription factors and microRNAs are the major families of gene regulators. Recent studies have suggested that these two kinds of regulators share similar regulatory logics and participate in cooperative activities in the gene regulatory network; however, their combinational regulatory effects and preferences on the protein interaction network remain unclear.

**Methods:**

In this study, we constructed a global human gene regulatory network comprising both transcriptional and post-transcriptional regulatory relationships, and integrated the protein interactome into this network. We then screened the integrated network for four types of regulatory motifs: single-regulation, co-regulation, crosstalk, and independent, and investigated their topological properties in the protein interaction network.

**Results:**

Among the four types of network motifs, the crosstalk was found to have the most enriched protein-protein interactions in their downstream regulatory targets. The topological properties of these motifs also revealed that they target crucial proteins in the protein interaction network and may serve important roles of biological functions.

**Conclusions:**

Altogether, these results reveal the combinatorial regulatory patterns of transcription factors and microRNAs on the protein interactome, and provide further evidence to suggest the connection between gene regulatory network and protein interaction network.

## Background

A gene regulatory network (GRN) is a comprehensive collection of regulatory relationships that controls the global gene expression and the dynamics of protein output in a living cell [1-6]. These regulatory relationships may be derived from different layers in the gene regulatory system. Hence, a GRN can be roughly separated into two major levels: the transcriptional and the post-transcriptional levels.

At the transcriptional level, a class of DNA-binding proteins, known as transcription factors (TFs), plays a major role in regulating gene expression. By binding to specific regions of DNA sequences, TFs can control the transcription activities of target genes, thus regulating the production of mRNA transcripts [7-9]. Since it has been widely believed that TFs are the primary regulators of gene expression, previous research on GRNs has mainly focused on the regulatory relationships at the transcriptional level [5,10,11]. However, there is increasing evidence suggesting that, at the post-transcriptional level, microRNAs (miRNAs) may also contribute to modulation of gene expression on a large scale [1-3]. miRNAs are small non-coding, single stranded RNAs of ~22 nucleotides in length that are abundantly found in eukaryotic cells [1-3]. By binding to complementary sequences (a.k.a. miRNA binding-sites) on target messenger RNA transcripts (mRNAs), miRNAs can trigger translational repression or gene silencing, thus regulating the expression of their target genes at the post-transcriptional level [12,13]. In recent years, miRNAs have been reported to control many biological processes, such as development, differentiation, growth, and even cancer development and progression [1-3]. Therefore, it has become critical to construct an integrated GRN that comprises both transcriptional and post-transcriptional regulatory interactions.

Similar to other biological networks, a GRN usually consists of several types of sub-network patterns known as network motifs, such as feedback and feedforward loops. Previous studies [5,10,11] have shown that certain types of network motifs are more overrepresented in GRNs[14]. These network motifs, such as feedback loops and co-regulation, are found to play pivotal roles in gene regulation [15-17]. For example, in *E. coli*, ~35% TFs participate in negative autoregulation motifs which can significantly speed up the transcriptional response time [15] and smooth the fluctuations of protein expression [16]. In addition to TFs, miRNAs may also form specific network motifs in the GRN. Previous studies [17-20] investigating the co-regulation between miRNAs and TFs found a variety of significant network motifs overrepresented in the co-regulation network, suggesting that the gene regulatory system requires close cooperation between transcriptional and post-transcriptional layers. These studies each proposed that the network motifs might be used as building blocks in GRNs. In order to understand how these motifs in the GRN influence the downstream biological processes, further studies on the protein interactome are essential.

Proteins are the major functional units in living cells, and usually do not work alone. Protein-protein interactions (PPIs), formed by two physically interacting proteins, are fundamental to most biological processes. In addition, proteins are translated from mRNAs, and therefore their abundance may be affected by upstream miRNAs and TFs. Consequently, investigating the correlations between PPIs and their upstream regulators could facilitate the understanding of biological mechanisms within living cells. Recently, the correlations between miRNAs and PPIs have been investigated [21,22]. Liang and Li [21] revealed that proteins regulated by more miRNAs tend to possess higher degree, more interacting partners, in a protein interaction network (PIN). Furthermore, Hsu *et. al. *[22] provided a comprehensive analysis and suggested that miRNAs could influence specific biological processes through regulating a small number of selected proteins in a PIN, such as hub and bottleneck proteins. These studies have revealed some connection principles between upstream regulators and downstream PINs. However, the specifics of the cooperation between TFs and miRNAs and their combinational regulatory effects on human PINs remain unclear.

**Figure 1:**
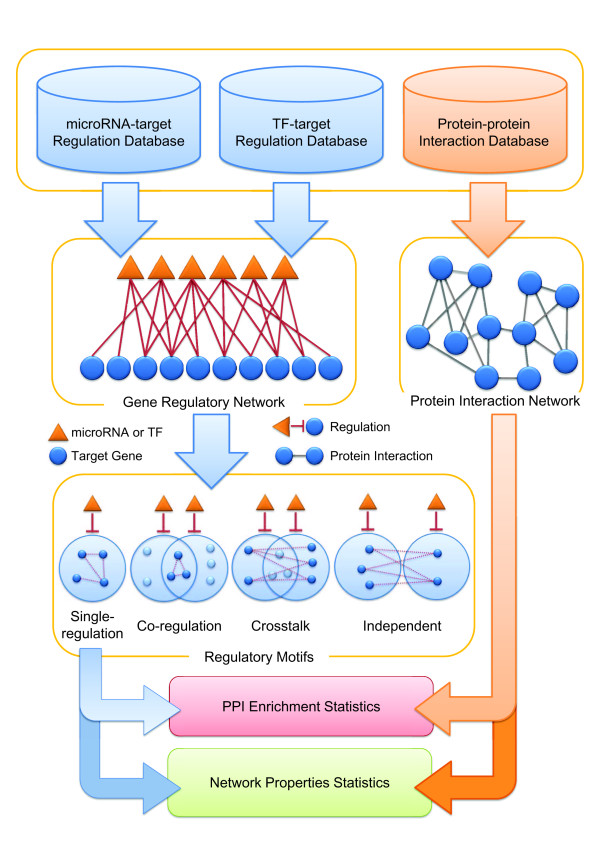
**The procedure for screening and analyzing the 4 types of regulatory motifs**. The GRNs was combined from TF and miRNA regulatory relationships. According to the types of synergistic regulations between regulators, 4 types of regulatory motifs were screened, single-regulation, co-regulation, crosstalk, and independent. Then, human PINs were utilized to elucidate the significance of the correlations between these 4 types of regulatory motifs and PPIs

In this study, we firstly collected human TF and miRNA regulatory relationships and integrated them into a global GRN. Next, we imported the human protein interactome into the GRN and screened the integrated network for four pre-defined regulatory patterns (Figure 1). Among the four patterns, the crosstalk was found to have the most enriched PPIs interconnected in their downstream regulatory target sets. Notably, the observed correlation between PPIs and the crosstalk motif has not been previously reported. Further investigation into the topological properties of the crosstalk motifs also revealed that they might serve important roles of biological functions. We thus propose that the crosstalk motifs may play significant roles in PINs, which may have important downstream effects on several biological processes in living cells via regulating corresponding PPIs.

## Methods

### Gene regulatory networks and protein-protein interactions

To construct the human GRN for our analysis, we collected TF and miRNA regulatory relationships from three online databases: TRED (Transcriptional Regulatory Element Database) [23], UCSC genome browser at http://genome.ucsc.edu/, and TargetScanHuman (release 6.0, November 2011) [24-27]. TRED contains transcriptional regulation information from experimental evidence and computational prediction. We collected 6,764 transcriptional regulation relationships between 133 TFs and 2,937 target genes from TRED. Additionally, we obtained the conserved binding sites of 125 TFs from UCSC genome browser. To identify the targets of these 125 TFs, the annotations of 21,368 human genes were downloaded from UCSC genome browser. We assigned a target gene to a TF if its promoter region (1000 bp upstream and 500 bp downstream of the transcription start site) covered at least one conserved binding site of the TF [28]. After this process, we identified 52,301 regulations between 125 TFs and 12,383 targets. Then, the union of these two transcriptional regulatory networks from TRED and UCSC was considered as the GRN of transcriptional level in this study, containing 58,711 regulations between 211 TFs and 13,402 targets. For miRNA target prediction programs, previous study had noticed that TargetScan possessed relatively higher precision and sensitivity than other programs [29]. We collected 144,490 post-transcriptional regulatory relationships between 153 miRNA families and 11,161 target genes with conserved binding sites of corresponding miRNAs. Next, these regulatory relationships collected from the three databases were merged together to construct our global GRN, in which nodes represent regulators (TFs/miRNAs) or target genes/proteins, and edges represent the regulatory relationships between regulators and targets.

Human PPI data were obtained from HPRD (Human Protein Reference Database) [30], which contained experimentally validated physical interactions among human proteins. In this study, we collected 37,080 interactions between 9,465 proteins.

Considering the incompleteness of current human PPI data, we performed an analogous analysis with an expanded PIN, a union of BioGRID [31] and HPRD PPI data, to verify our results. Additionally, since limited reproducibility of miRNA target prediction has also been reported [32-35], we further independently repeated our study with another miRNA target prediction database, miRBase [36], to confirm the robustness of our conclusions.

### Regulatory motif screening and analysis

We screened four types of regulatory motifs from GRN: single-regulation, co-regulation, crosstalk, and independent, considering possible synergistic regulation between regulators. These regulatory motifs are depicted in Figure 1. The synergistic regulation defined here is determined by whether the regulators shared at least two common targets. A single-regulation motif consists of one regulator and its targets. The other three motifs consist of two regulators. The co-regulation motif is formed by two synergistic regulators and their shared targets. The crosstalk motif is formed by two synergistic regulators and their private (non-shared) target sets. The independent motif contains two non-synergistic regulators and their respective target sets.

Next, the PPI enrichment for each type of regulatory motif was analyzed. Specifically, for single-regulation, PPIs between every paired target genes were analyzed; for co-regulation motifs, only PPIs between common target genes were analyzed; and for crosstalk and independent motifs, only PPIs between two private target gene sets were analyzed. Additionally, the PPI enrichment analysis was performed from two directions: top-down and bottom-up. In the bottom-up model, genes were firstly classified into four categories analogous to four types of regulatory motifs, and each category was provided with one significance score. In the top-down model, significance scores were assigned to each regulator (for single-regulation motif) or to every pair of regulators (for the other three types of motifs). In this study, a significance score was defined as the *z-*score (standard score) derived from statistical analysis (Methods in Additional file 1). Furthermore, we also analyzed the significance of several selected network properties (Methods in Additional file 1) for each type of regulatory motif based on similar approaches adopted in the PPI enrichment analysis. The procedures of regulatory motif screening and analysis are depicted in Figure 1. In addition, the functional enrichment analysis of crosstalk motifs was performed to investigate the underlying biological roles for crosstalk motifs in human PINs (Methods in Additional file 1).

## Results

### Gene regulatory network properties

**Figure 2:**
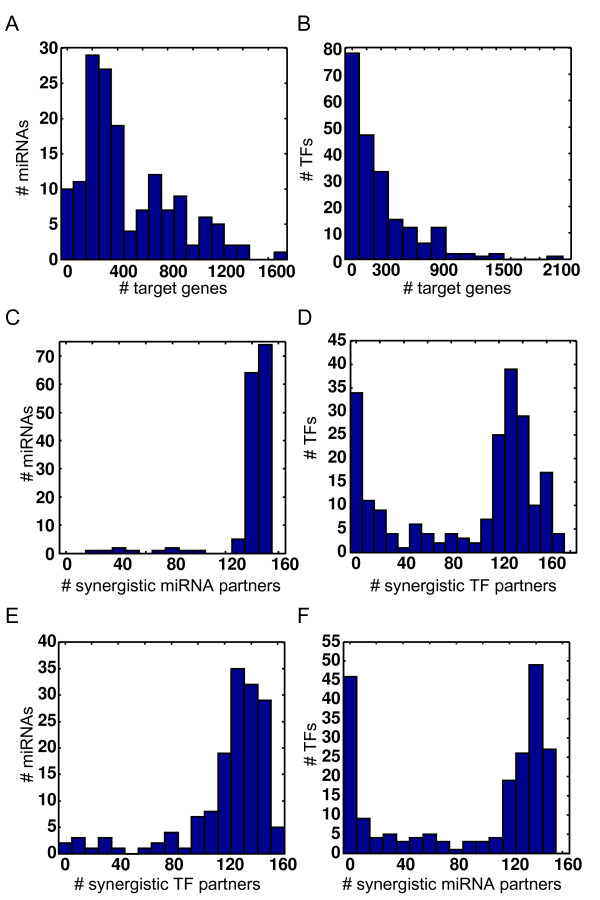
**Properties of gene regulatory networks**. The distributions of the number of target genes and synergistic partners for miRNAs and TFs.

In order to provide a global view of human GRN, both transcriptional and post-transcriptional regulations were analyzed jointly (the global GRN) and respectively (local GRNs) in this study. Within the local GRNs, TF- and miRNA-regulation displayed similar patterns of distribution with respect to the number of target genes (Figure 2A and 2B). Most TFs and miRNAs possessed relatively fewer targets, and only a small fraction of TFs and miRNAs possessed a large number of targets.

To investigate the synergistic relationships between regulators, we further analyzed the distributions of the number of synergistic partners of miRNAs and/or TFs (Figure 2C-F). Herein, we defined two regulators as having a synergistic relationship if they shared at least two common targets. Most TFs and miRNAs have at least one synergistic TF and/or miRNA partner. In other words, they tended to form synergistic regulations with other regulators. Although we noticed that a small fraction of TFs did not form synergistic regulations with other TFs or miRNAs (Figure 2D and 2F), this could be due to the lack of sufficient TF-regulation information.

### PPI enrichment of regulatory motifs

From the global GRN, we screened four types of regulatory motifs: single-regulation, co-regulation, crosstalk, and independent. Next, the PPI enrichment of regulatory motifs was investigated from two directions: top-down and bottom-up. Based on the combinations of regulators, the regulatory motifs of TF-TF, miRNA-miRNA, and TF-miRNA were analyzed separately.

**Figure 3:**
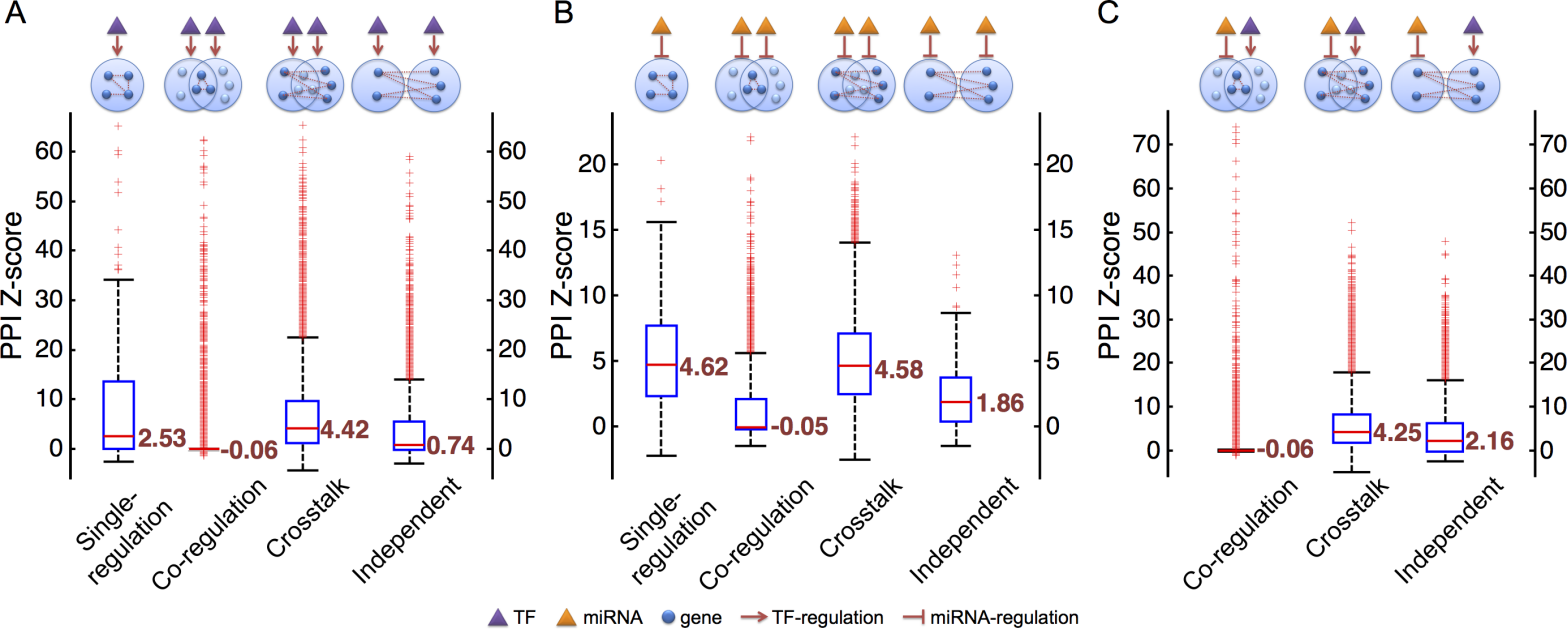
**PPI *z*-scores of regulatory motifs**. The box-plots of PPI *z*-scores for regulatory motifs regulated by different combinations of regulators: (A) TF-TF, (B) miRNA-miRNA, and (C) TF-miRNA. The numbers next to the red line in the box-plot represent the median of PPI *z*-scores.

In the top-down analysis, the significance scores of the motifs regulated by single TF or paired-TF are described in Figure 3A. The single-regulation and crosstalk motifs showed significantly enriched PPIs between regulated genes, while the co-regulation and independent motifs did not. Similarly, motifs regulated by miRNA or paired-miRNA also showed significantly enriched PPIs between regulated genes involved in the single-regulation and crosstalk motifs, but co-regulation and independent did not (Figure 3B). The single-regulation motif has been reported to be highly correlated with PPIs [21,22], which is consistent with our results. However, although correlations between PPIs and the co-regulation motif have also been reported and well-discussed [37,38], our analysis was inconsistent with these findings. For co-regulation motifs, the tested sample is the common targets between two regulators. Thus, we doubted that the insignificance of the co-regulation motifs was due to limited sample sizes of the common targets. To test this hypothesis, we gradually adjusted the threshold of synergistic regulation (i.e. the minimum number of shared targets) for co-regulation motifs. As the threshold increased, the *z-*scores of PPI enrichment of the co-regulation motifs also increased (Figure S1 in Additional file). This result suggested that the significance scores of the co-regulation motifs were truly affected by the sample size. In other words, the PPI enrichment would emerge if we adopted a stricter definition of co-regulation which means more common targets. For example, if the threshold of the synergistic regulation between TFs increases to 40 targets, the significance score would be elevated to 2. This result suggested that regulator pairs with common targets tend to regulate private targets with PPIs. The motifs regulated by TFs and miRNAs simultaneously were also investigated. Consistent with the results of the TF-TF and miRNA-miRNA analyses, the crosstalk motifs showed a significant correlation with PPI enrichment, while the co-regulation motifs did not (Figure 3C). Similarly, significance scores of the co-regulation motifs regulated by TF-miRNA combinations were affected by the sample size as well (Figure S1 in Additional file).

**Figure 4:**
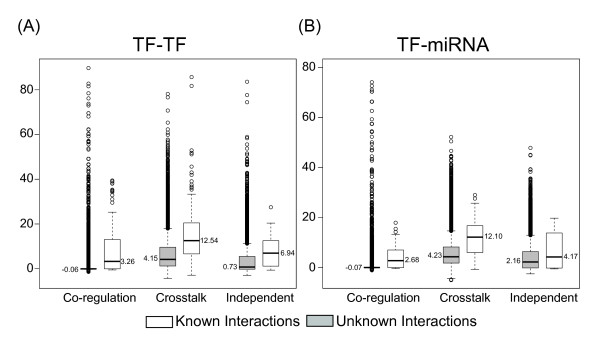
**PPI *z*-scores of regulatory motifs regulated by known and unknown interacting TF-TF/TF-miRNA pairs**. The box-plots of PPI *z*-scores for regulatory motifs regulated by different combinations of regulators: (A) TF-TF and (B) TF-miRNA. The numbers next to the black lines in the box-plots represent the median of PPI *z*-scores.

Considering the reported TF-TF and TF-miRNA interactions [20], we divided each proposed motif into two subcategories, with or without known interactions of the regulator pairs. Motifs regulated by interacting regulator pairs displayed higher PPI *z-*scores than those without known interactions (Figure 4). Notably, the crosstalk motifs showed the highest *z-*scores. This observation further confirmed our suggestions that regulatory motifs with synergistic relationships tend to regulate genes with PPIs, especially for crosstalk motifs.

For the bottom-up analysis, we investigated the PPI enrichment of genes sets in four types of regulatory motifs. The significance score and corresponding coverage of each type of regulatory motifs are presented in Table 1. In all combinations of regulator pairs, target genes of the single-regulation and co-regulation motifs showed significant PPI enrichment, but those of the independent motifs showed significantly insufficient PPI contents. This suggested that the targets of the single-regulation and co-regulation motifs preferred to form PPIs, but those of the independent motifs did not. With respect to the crosstalk motifs, targets regulated by TF-TF, miRNA-miRNA, and TF-miRNA pairs showed insignificant PPI enrichment. Here, we noticed that the coverage of the crosstalk motifs was much higher than the other three types of motifs, at almost 100%. This high coverage means that the tested sample is nearly identical to the whole population; therefore, the enrichment could be insignificant owing to the loose definition of crosstalk motifs. To test this hypothesis, we removed those regulators whose target sizes were excessively larger than other regulators; in other words, we removed the top outliers from the target-size distribution of regulators. Indeed, after this procedure, the PPI enrichment of crosstalk motifs emerged, and the results of other types of motifs remained the same (Table 1 Figure S2 in Additional file). In summary, genes under the control of regulatory motifs tend to form PPIs, except for those genes regulated by independent motifs.

**Table 1:** PPI *z*-score and the coverage of gene sets involved in regulatory motifs

	Single-regulation	Co- regulation	Crosstalk	Independent
	TF-TF			

*z*-score	62.17	62.10	-0.80	-27.32

coverage	16.19%	4.31%	96.65%	83.81%

*z*-score*	56.43	51.37	11.37	-15.14

coverage*	6.72%	0.92%	70.60%	93.28%

	miRNA-miRNA			

*z-*score	35.37	38.15	1.52	-20.79

coverage	25.68%	9.14%	96.60%	74.32%

*z*-score*	34.74	32.16	14.37	-12.08

coverage*	10.79%	2.15%	69.08%	89.21%

	TF-miRNA			

*z*-score	-	62.44	-0.09	-55.91

coverage	-	6.65%	97.42%	73.52%

*z-*score*	-	47.94	15.71	-33.91

coverage*	-	1.18%	70.49%	88.31%

*The asterisk represents the filtered values. The upper limit of target gene size for filtration of TF-TF, miRNA-miRNA, and TF-miRNA motifs is 781, 789, and 789, respectively.

*The coverage represents the ratio of gene pairs in regulatory motifs to all possible gene pairs in HPRD PIN.

According to the results of the top-down and the bottom-up analyses, we came to three conclusions: 1) the single-regulation motifs tend to regulate genes with PPIs. 2) Regulatory motifs with synergistic relationships (i.e. co-regulation and crosstalk) favor gene regulation with PPIs, especially for crosstalk motifs. 3) Gene pairs regulated by independent regulators (i.e. without synergistic relationships), in contrast, show no preference to form PPIs.

### Regulatory motifs tend to regulate pivotal proteins in PIN

Genes encoding proteins with meaningful network properties in PINs have been proposed to play very important roles in living cells [39-44]. PPI enrichment analysis suggested that the single-regulation, co-regulation, and crosstalk motifs are highly correlated with PPIs. Herein, we further investigated the network properties of target genes (Methods in Additional file 1) involved in these three types of motifs. The *z*-scores of each of the network properties for regulatory motifs are summarized in Table 2 (more details in Figure S3 in Additional file). Network properties can be classified into two categories: 1) for individual genes--degree and closeness centrality; 2) for gene sets--density, clique level, and path length.

**Table 2:** *Z*-scores of each network properties for regulatory motifs

Motif	Single-regulation		Co-regulation			Crosstalk		
**Regulator (s)**	**TF**	**miR**	**TF**	**miR**	**TF-miR**	**TF**	**miR**	**TF-miR**

Degree	1.52	1.29	1.95	0.72	0.83	2.35	1.80	1.59

Closeness	1.55	1.86	2.02	1.22	1.26	2.45	2.73	2.12

Density	2.31	3.88	1.60	0.96	1.28	13.74	4.33	5.27

Clique level	1.51	1.46	1.53	1.19	1.00	2.14	2.02	1.63

Path length	-15.58	-24.10	-10.25	-6.66	-7.52	-21.27	-38.25	-50.47

*For single-regulation motifs, there are two types of regulators, TF and miRNA (miR). For co-regulation and crosstalk motifs, there are three types of regulators, TF-TF, miRNA-miRNA, and TF-miRNA. Here, we used TF and miR to represent TF-TF and miRNA-miRNA respectively. Path length represents the characteristic path length of the gene set.

With respect to individual genes, most regulatory motifs tend to regulate those genes with higher degree and closeness centrality (*z*-score > 1). Degree represents the connectivity of proteins in a PIN, and closeness centrality represents how close proteins are to the center of a PIN. These results suggested that the regulatory motifs tend to regulate hub and central proteins. On the other hand, most regulatory motifs tend to regulate those gene sets with higher density (*z*-score > 1), larger clique levels (*z*-score > 1), and significantly shorter path lengths (*z*-score < -2). Density provides a quantitative measure of how tested gene sets group together to form a community in a PIN, clique level represents the level of maximal clique in which a tested gene can join, and path length describes how close tested proteins are to each other in a PIN. Briefly, these three network properties were usually used to evaluate the modularity of tested proteins. Hence, the results presented here imply that the regulatory motifs tend to control those proteins that form biological communities. Notably, the crosstalk motifs showed more significant *z-*scores than other types of regulatory motifs, suggesting they play more roles that are important in PINs.

### Biological processes of the crosstalk motifs

**Figure 5:**
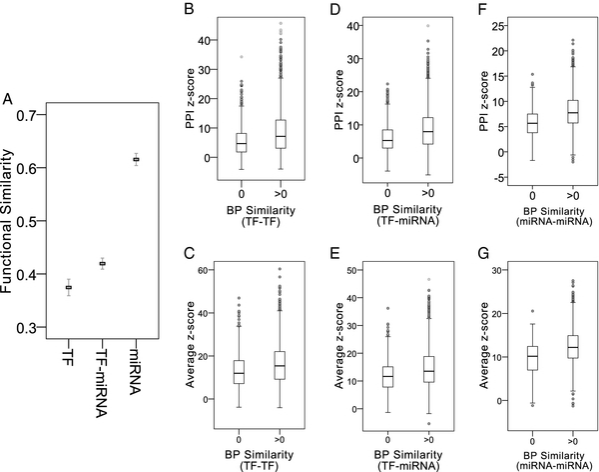
**Functional features of the crosstalk motifs**. (A) The functional similarity between two regulators in the crosstalk motifs. The rectangular markers indicate the average values and error bars indicate the 95% confidence intervals. (B)--(G) Comparison of PPI *z*-score and average network property *z*-score between the crosstalk motifs with zero and non-zero functional similarity. Average *z*-score represented the averaged network property *z*-score.

After investigating the PPI enrichment and network properties of the screened regulatory motifs, we noticed that the crosstalk motifs played a pivotal role in human PINs, and hence further studied their biological processes. First, we analyzed the functional similarity between two regulators. The results are shown in Figure 5A. Functional similarities between regulator pairs of crosstalk motifs, ranked in descending order, are as follows: miRNA-miRNA, TF-miRNA, and TF-TF (avg., 0.62, 0.42, and 0.37, respectively). Lower functional similarity in TF-TF and TF-miRNA pairs might reflect the dominant positions of TFs in global regulatory system (i.e. at the transcriptional level) [45]. Contrarily, higher functional similarity in miRNA-miRNA pairs might be due to the downstream positions of miRNAs in the global regulatory system (i.e. at the post-transcriptional level) [45]. In addition, we observed that a notable proportion of crosstalk motifs are with zero functional similarity (TF-TF: 29%; TF-miRNA: 22%; miRNA-miRNA: 14%, Figure S4 in additional file), i.e. no common enriched functions between two regulators. To investigate this observation further, we compared the PPI *z*-scores and the averaging network property *z*-scores of crosstalk motifs with zero versus non-zero functional similarity. For all regulator combinations, the crosstalk motifs with non-zero functional similarity showed significantly higher PPI *z*-scores and network property *z*-scores than those with zero functional similarity (Figure 5B-G and Table 3). Therefore, the zero functional similarity might be due to the lack of PPIs between regulated private targets involved in the crosstalk motifs. This result suggests that the functional synergistic regulations of the crosstalk motifs could be based on the PPIs between regulated private target genes, highlighting the functional features of the crosstalk motifs.

**Table 3:** PPI and network property *z*-score of crosstalk motifs with zero and non-zero functional similarity

	TF		TF-miRNA		miRNA	
***z*-score**	**PPI**	**Network**	**PPI**	**Network**	**PPI**	**Network**

0	5.71	12.96	6.15	11.81	5.68	9.63

> 0	8.89	16.43	8.68	14.59	8.03	12.38

*p*-value	2.07E-15	1.89E-11	8.29E-29	2.31E-13	3.87E-42	2.02E-24

*P-values were derived from Wilcoxon rank-sum test. TF and miRNA represented TF-TF and miRNA-miRNA combinations respectively. Network represented the average *z*-score of the network properties. *Z*-scores in this table were averaged.

**Figure 6:**
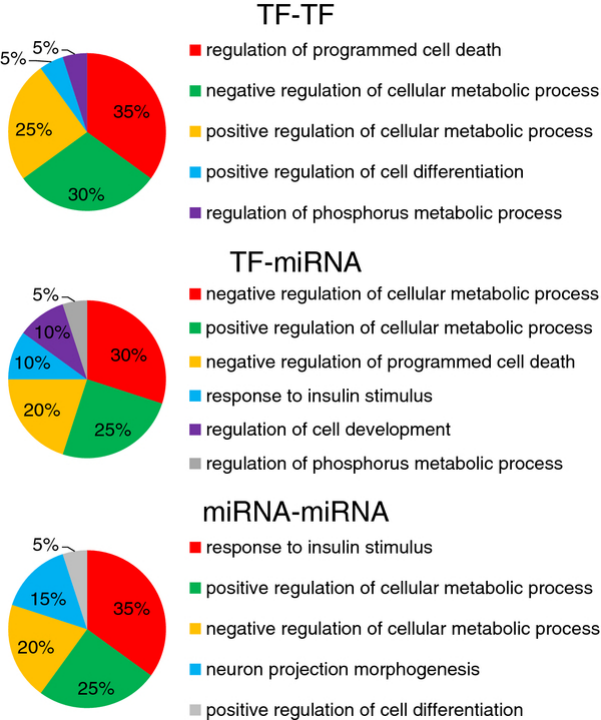
**Crosstalk Functions of the crosstalk motifs**. The summary of enriched crosstalk functions in the crosstalk motifs.

We then studied the underlying biological processes between private targets of crosstalk motifs (Methods in Additional file 1). For each combination of regulator pair, we selected top 20 biological processes ranked by proportions of involved motifs, respectively (Additional file 1 Figure S5 - S7). These biological processes covered nearly all the crosstalk motifs (TF-TF: 98.02%; TF-miRNA: 100%; miRNA-miRNA: 99.88%). Figure 6 shows a summary of these processes. The majority of selected processes for all three types of regulator pairs are associated with positive/negative regulation of cellular metabolic process. Notably, TF-TF crosstalk motifs also favor the processes associated with regulation of programmed cell death (apoptosis); miRNA-miRNA ones favor those with response to insulin stimulus; and TF-miRNA with both. These results not only displayed the functional homogeneity between regulators of crosstalk motifs, but also demonstrated the difference between TF and miRNA at regulatory level.

**Figure 7:**
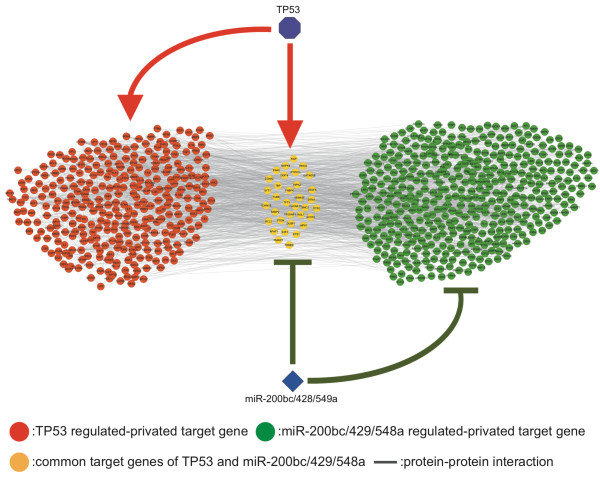
**The TP53-miR-200bc/429/548a crosstalk motif**. The visualization of the TP53-miR-200bc/429/548a crosstalk motif. There are 1,918 PPIs between target genes in this motif. 25.02% and 25.50% PPIs formed intra-connections within TP53 and mi-200bc/429/548a regulated-private target genes, respectively; 39.05% PPIs formed inter-connections between two private target sets.

We further inspected the potential biological processes of the TP53-miR-200bc/429/548a crosstalk motif (Figure 7) as it possessed the highest PPI *z*-score. In this crosstalk motif, there are 1,918 PPIs between target genes: around 25.02% and 25.50% PPIs formed intra-connections within TP53 and miR-200bc/429/548a regulated-private target genes, respectively, and around 39.05% PPIs formed inter-connections between two private target sets. The enriched interconnected PPIs within these motifs might imply massive crosstalk between regulators in their downstream regulatory pathways. PPIs between private targets of TP53-miR-200bc/429/548a were enriched in positive/negative regulation of cell death, response to insulin stimulus, epidermal growth factor receptor signaling pathway, toll-like receptor signaling pathway, positive regulation of cell differentiation/proliferation, regulation of protein kinase activity, protein phosphorylation, and regulation of cell migration. TP53 is a well-studied cancer-related gene which encodes the tumor-suppressor protein p53 [46-48], and miR-200bc/429/548a has been reported to be significantly down-regulated in and related to several cancers [49-53]. For example, Shimon Y. *et al*. reported that miR-200bc/429/548a suppressed the ability of tumor formation driven by human breast cancer stem cell in vivo [50]; and Hu X. *et al*. reported that the overexpression of miR-200bc/429/548a could inhibit the cell migration of ovarian cancer cell and thus suggested that this miRNA should be strongly associated with cancer recurrence and overall survival [51]. Therefore, the TP53-miR-200bc/429/548a crosstalk motif might be a potential cancer-related regulatory motif.

## Discussion and conclusions

In this study, we incorporated miRNAs into a traditional GRN to investigate the correlations between PPIs and regulatory motifs formed by miRNAs, TFs, and target proteins/genes. The regulatory motifs were classified into four types: single-regulation, co-regulation, crosstalk, and independent. Traditionally, random sampling methods are usually applied to evaluate the significance of PPI numbers among a group of proteins, but this is very time-consuming. In addition, random sampling is not suitable for analyzing complicated regulatory networks, because the whole process should be redesigned for different motif members. In order to improve the efficiency of the evaluation process without loss of general applicability, we calculated the significance of PPI enrichment for different motifs based on the Bernoulli distribution; in other words, we regarded PPI gain and lost as a Bernoulli process. This allowed the whole evaluation process to be kept under constant time (O(1)).

Among the four types of motifs, the strong correlation between single-regulation and PINs has been well-discussed [21,22], and a correlation with the co-regulation type has also been reported [37,38]. Single-regulation motifs analyzed here showed consistent conclusions with previous studies. Our investigation into co-regulation motifs has further provided complementary analysis and given insights that have not been addressed in any previous studies. More importantly, we proposed that the third type of motif -- the crosstalk motif -- could be another prominent pattern in GRNs. Crosstalk motifs were defined as the private target gene sets of two corresponding regulators, TFs and/or miRNAs, which shared at least two targets. In human PINs, crosstalk motifs were significantly enriched in PPI contents and network properties. To summarize the analysis of network properties, crosstalk motifs displayed several features: 1) high degree, 2) high closeness, 3) high density, 4) high clique level, and 5) short characteristic path length. In PINs, proteins with a high degree are usually called "hub proteins", those with high closeness centrality are usually called "central proteins", and those with high density, short characteristic path length, and high clique level are usually called "modular proteins". Therefore, the regulators which participate in crosstalk motifs tend to regulate hub proteins, which are usually more essential than non-hub proteins [39-41], and modular proteins, which usually form important protein complexes or modules in human PINs [42-44]. Additionally, we investigated the enriched functions of the crosstalk motifs. For all three types of regulator pairs, the majority of enriched crosstalk functions are associated with positive/negative regulation of cellular metabolic processes. Notably, miRNA-miRNA crosstalk motifs are not only associated with regulation-related functions, but also response to insulin stimulus. This is consistent with previous findings that miRNAs preferentially regulate downstream components, such as TFs, in signaling networks [19]. Moreover, we demonstrated the functional features within the crosstalk motifs with the highest PPI *z*-score and proposed a potential cancer-related motif, TP53-miR-200bc/429/548a. Consequently, this crosstalk motif might play an important role in living cells through regulating those essential or pivot proteins in human PINs.

Since our analysis relies on limited data sources from online databases to construct human PINs and GRNs, we carried out further examinations to test the robustness of our conclusions. With respect to miRNA regulation, all current online databases which provide predicted human miRNA targets still have room for improvement both in approach and performance [32-35]. Accordingly, we repeated our analysis with another database, miRBase [36], and were able to reach a consistent conclusion (Figure S8-S13, Table S1 and S2 in Additional file 1). Considering the incomplete and noisy human PPI data, we performed the same analysis with combined PPI data from HPRD and BioGRID [31] databases and also obtained consistent conclusions (Figure S14-S22, Table S3-S5 in Additional file 1). Therefore, these re-analyses provide further evidence to support the robustness of our conclusions. With ongoing efforts to improve the completeness of PPI data and GRNs, we will be able to further investigate and confirm the correlations between PPIs and regulatory motifs in the future.

In summary, we proposed a computational approach to investigate the significance of regulatory motifs formed by TFs/miRNAs and their corresponding targets in human PINs. With this approach, we screened four types of regulatory motifs, single-regulation, co-regulation, crosstalk, and independent, from human GRNs and investigated their correlations with PPIs. Among the four types of motifs, the crosstalk motif emerged as a potentially significant motif with important roles in PINs, which has not been previously reported. We suggested that this motif might play an important role in living cells because of its strong correlations with PPIs and significant network properties in human PINs.

## Competing interests

The authors declare that they have no competing interests.

## Authors' contributions

CCL, YJC, CYC, and HCH carried out the analysis, and drafted the manuscript. YJO, HFJ, and HCH conceived and directed the project, participated in the design and coordination of the study, and edited the manuscript. All authors read and approved the final manuscript.

## Supplementary Material

Additional file 1 **Supplementary methods, figures, and tables**. This file contains supplementary methods and results, and the repeat analysis for confirming the robustness of our results with different datasets, miRNA-target prediction and PPI data. Click here for file
