# Supplementary material for: Crosstalk between transcription factors and microRNAs in human protein interaction network
Source: BMC Syst Biol. 2012 Mar 13;6:18. doi: 10.1186/1752-0509-6-18 (PMC3337275; doi:10.1186/1752-0509-6-18)
Supplement: Additional file 1 — Supplementary methods, figures, and tables. This file contains supplementary methods and results, and the repeat analysis for confirming the robustness of our results with different datasets, miRNA-target prediction and PPI data. [file 1752-0509-6-18-S1.PDF]

Crosstalk between transcription factors and microRNAs in human protein interaction network

Chen-Ching Lin, Ya-Jen Chen, Cho-Yi Chen, Yen-Jen Oyang, Hsueh-Fen Juan, Hsuan-Cheng Huang

Additional File

Figures: Figure S1-S22

Tables: Table S1-S5

Methods

References

Figures

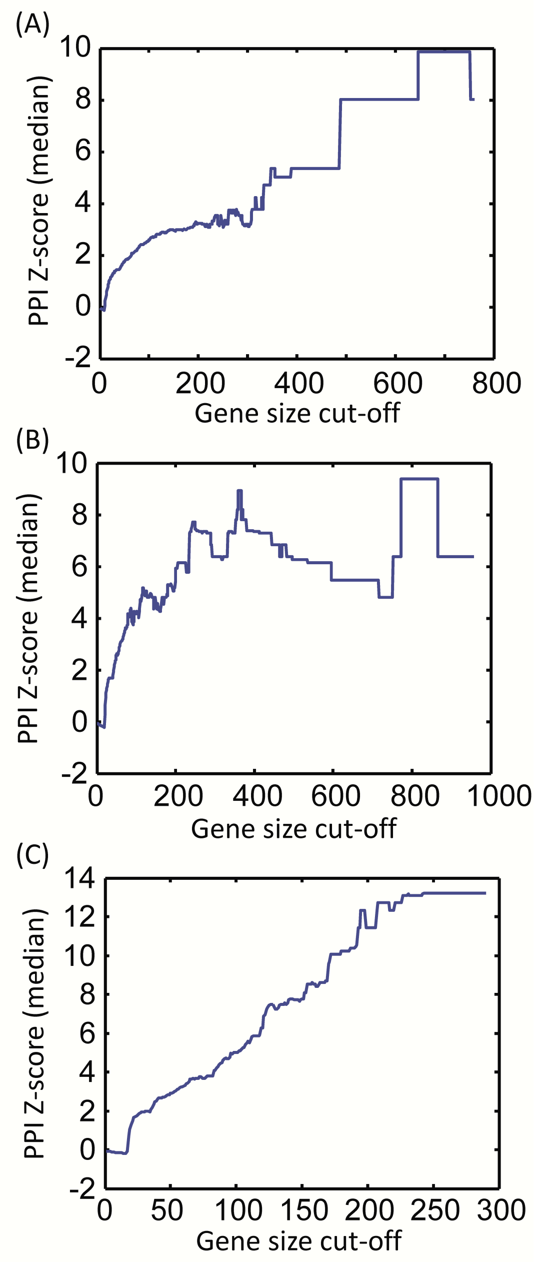

Figure S1. Correlation between PPI *z*-score and the cut-off of synergistic regulation between regulators.

Gene size means the number of the common targets between two regulators. As the lower cut-off increases, the z-score of PPI enrichment of the co-regulation motifs also increased. (A) TF-TF (B) miRNA-miRNA (C) TF-miRNA

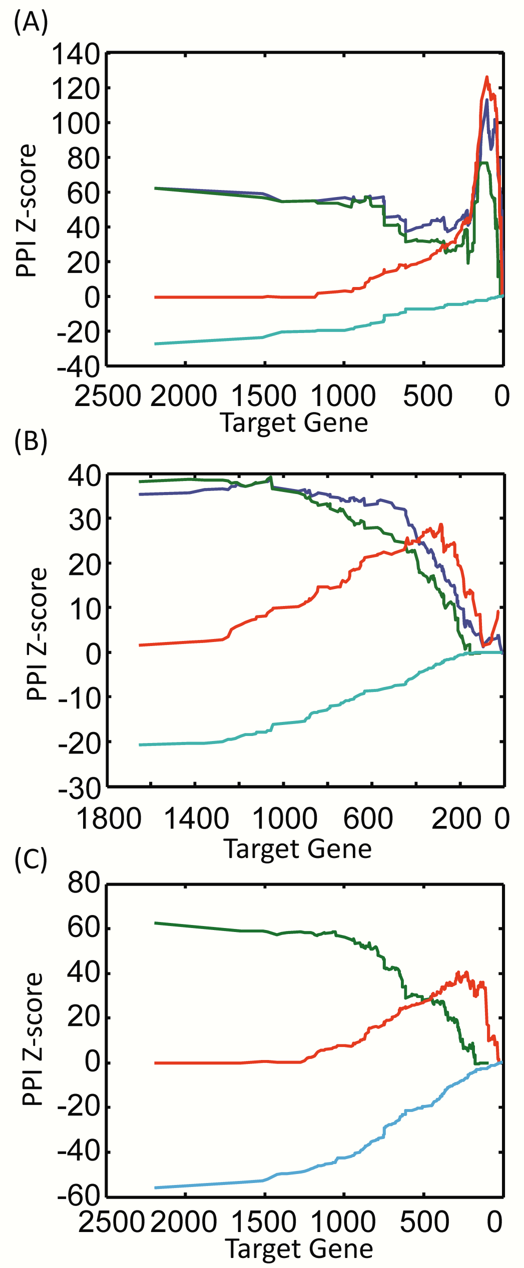

Figure S2. Correlation between PPI z-score and the number of the targets regulated by regulators.

While the upper limit of the target gene size (number) decreasing, the significance of PPI enrichment of gene pairs involved in crosstalk motifs increased. The decreasing trend of PPI z-score in the tail might be due to the small sample size. Blue: single-regulation; Green: co-regulation; Red: crosstalk; Light blue: independent. (A) TF-TF (B) miRNA-miRNA (C) TF-miRNA

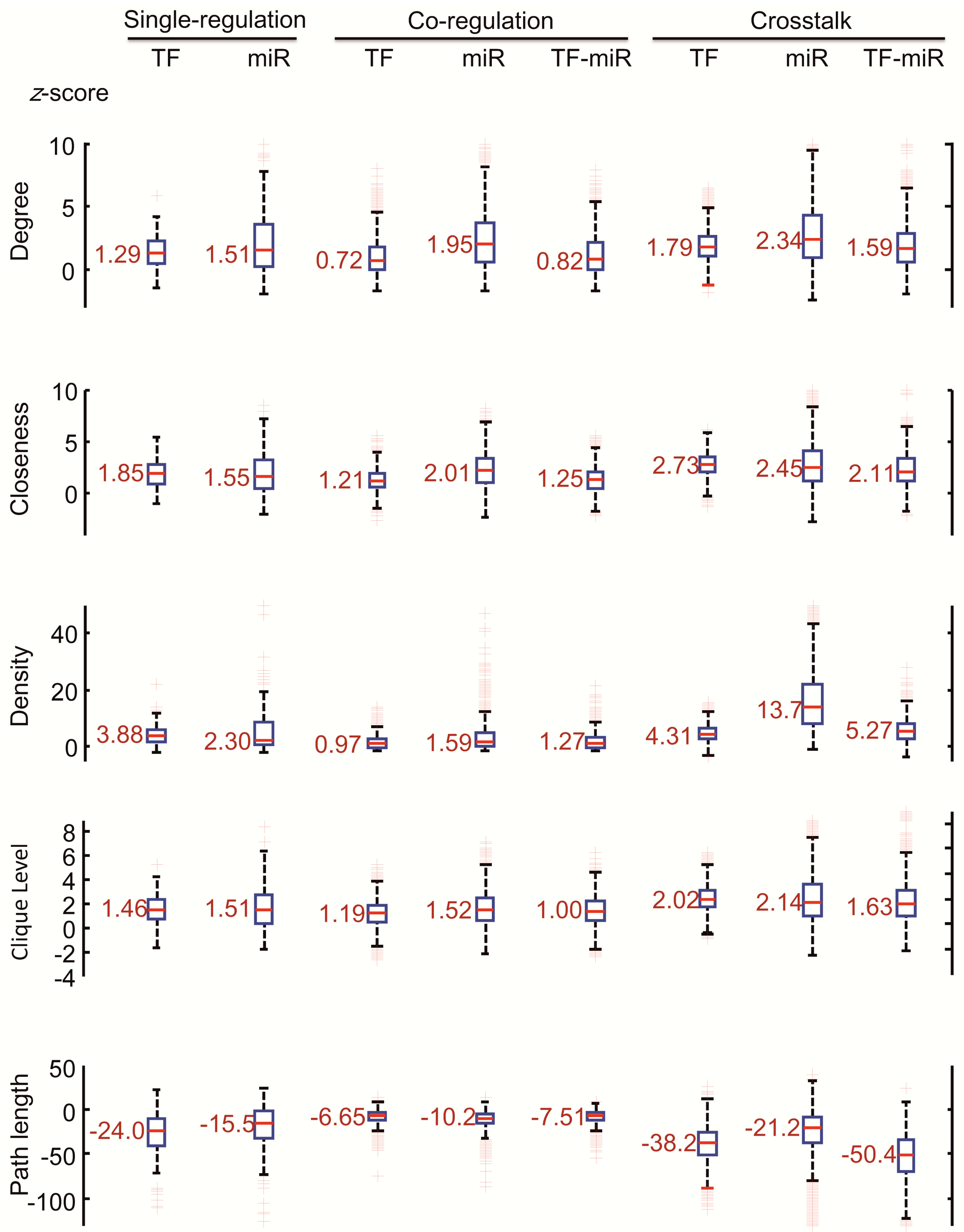

Figure S3. *Z*-score box-plots of network properties for regulatory motifs.

miR is the abbreviation for miRNA. For the co-regulation and crosstalk motifs, TF represents TF-TF pair and miR represents miRNA-miRNA pair. For all network properties, crosstalk motifs displayed the most significant value.

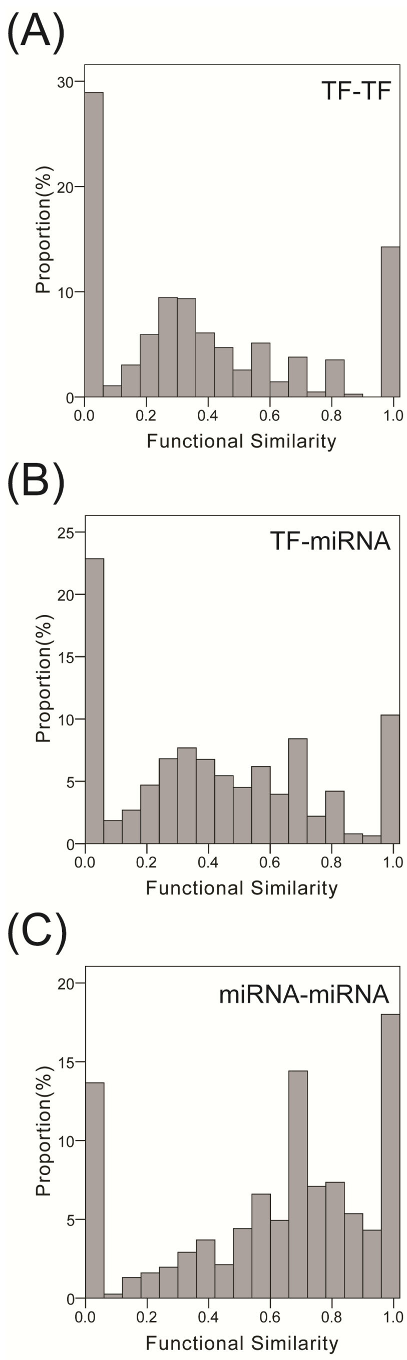

Figure S4. The distributions of functional similarity of crosstalk motifs.

miRNA-miRNA crosstalk motifs possessed high proportions of complete functional similarity. All crosstalk motifs displayed a certain amount of zero functional similarity (no common functions between two regulators). (A) TF-TF (B) TF-miRNA (C) miRNA-miRNA

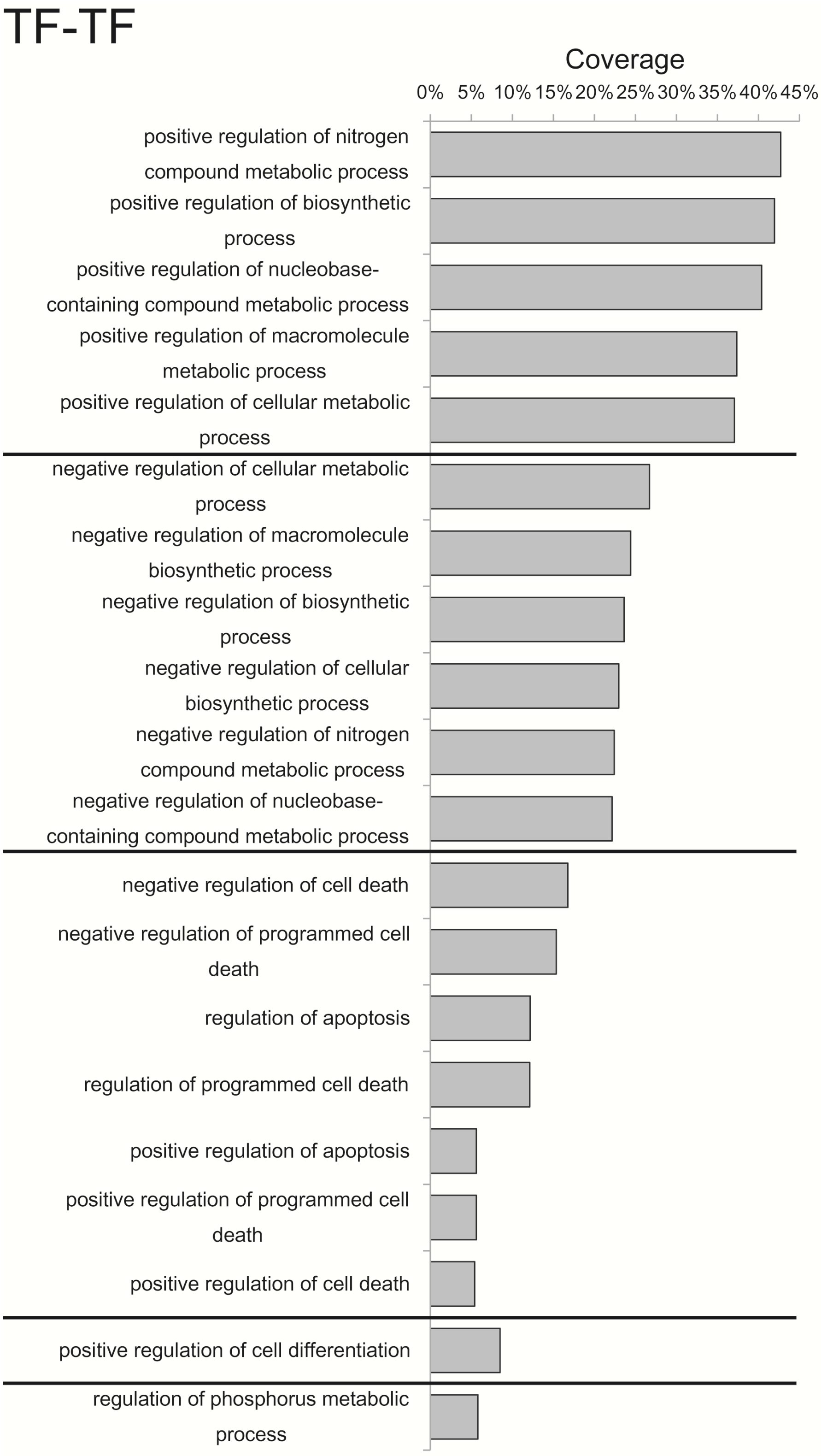

Figure S5. Top 20 biological processes that cover the highest proportions of TF-TF crosstalk motifs.

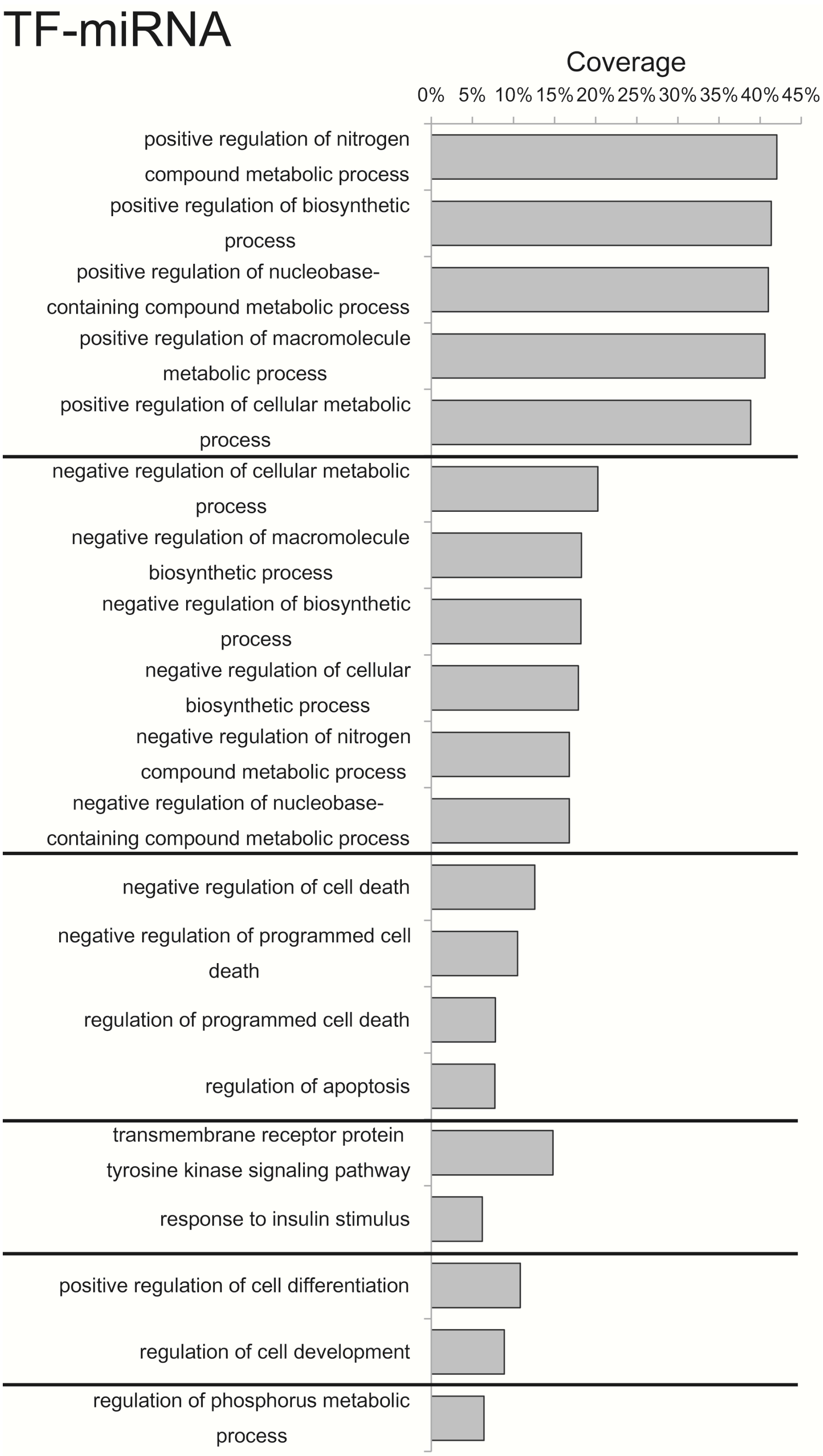

Figure S6. Top 20 biological processes that cover the highest proportions of TF-miRNA crosstalk motifs.

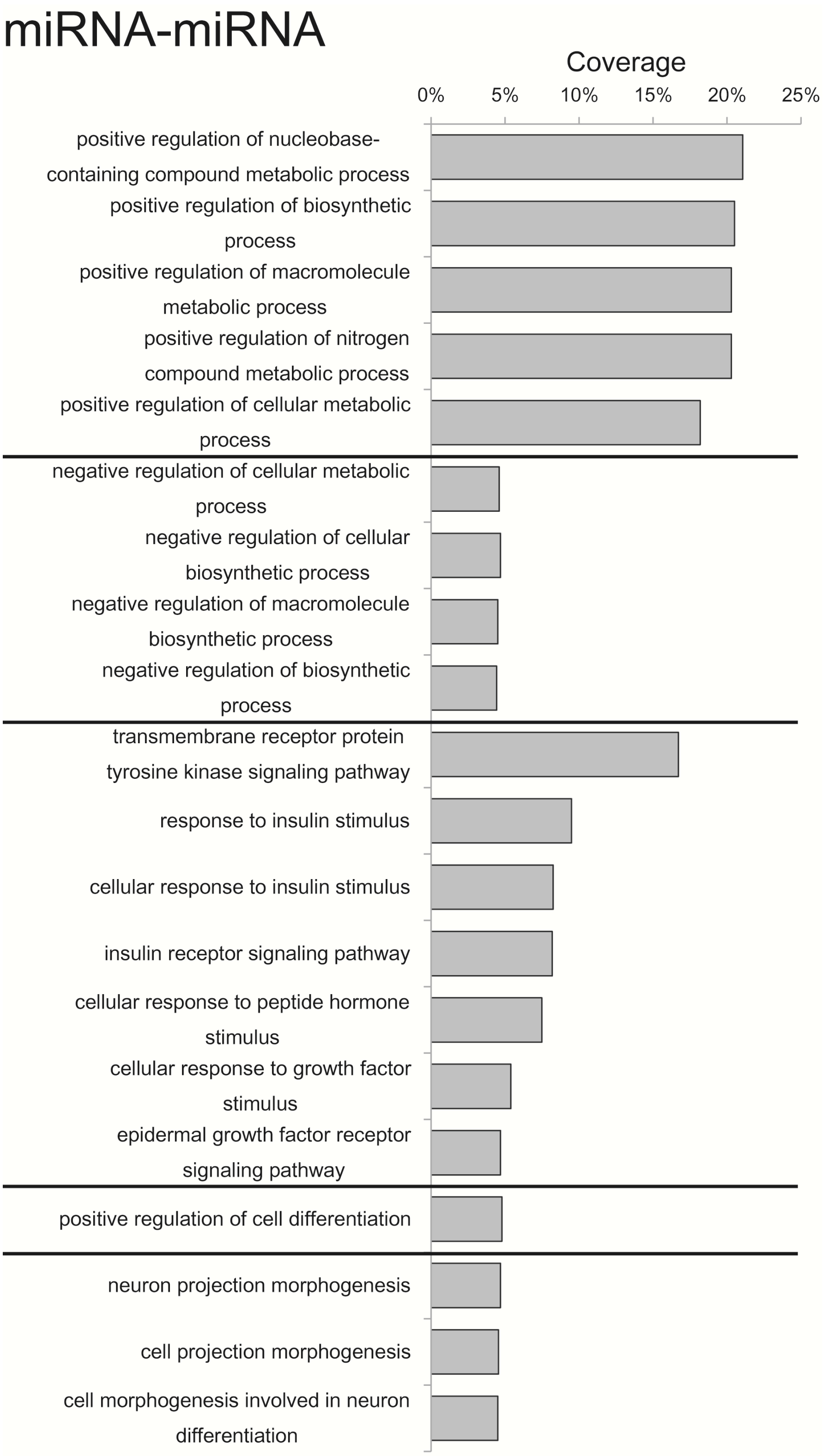

Figure S7. Top 20 biological processes that cover the highest proportions of miRNA-miRNA crosstalk motifs.

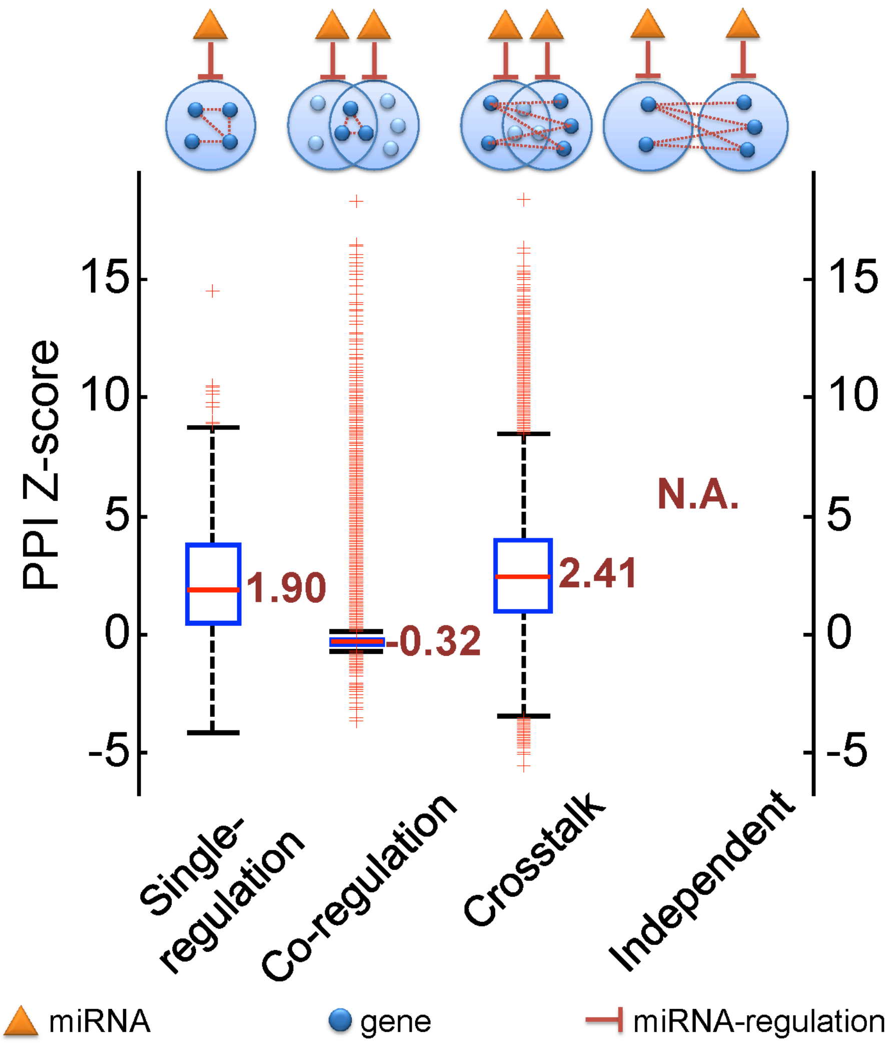

Figure S8. PPI enrichment analysis for 4 types of motifs of miRNA-miRNA pairs with miRBase dataset and HPRD PIN.

Consistent with the conclusions from the top-down analysis with TargetScan dataset, crosstalk motif displayed the most significant correlation with PPI. Since all the microRNAs (miRNAs) in miRBase shared at least one target with other miRNAs, the independent motif was non-available.

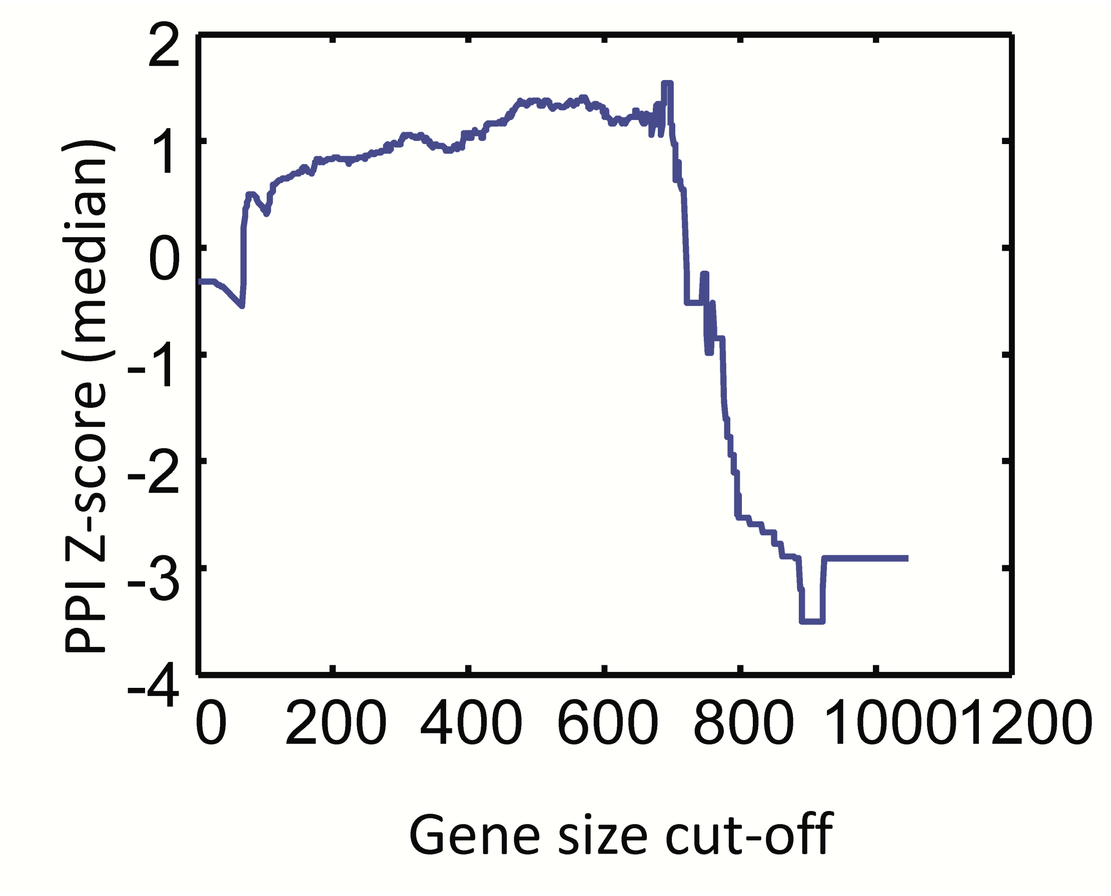

Figure S9. Co-target size filtration for co-regulation motifs of miRNA-miRNA pairs with miRBase dataset and HPRD PIN.

Consistent with the conclusions from the top-down analysis with TargetScan dataset, the z-score of co-regulation motif displayed a positive correlation with the cut-off of the co-target gene size (number). When the lower cut-off of the gene size went larger, the size of the tested samples was getting more and more similar with population became smaller. Therefore, the PPI z-score decreased became unpredictable in the tail. In other words, with the lower cut-off of co-target gene size (number) increasing, the significance of PPI enrichment of co-regulation motifs also increased.

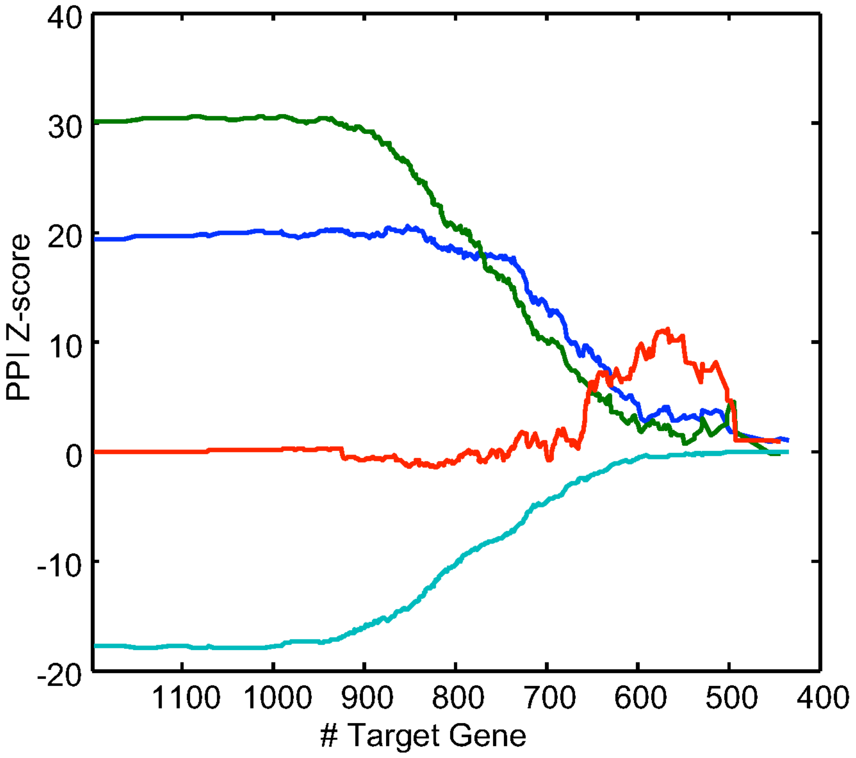

Figure S10. The correlation between the number of target genes and the z-score of PPI enrichment by using miRBase dataset and HPRD PIN.

Consistent with the conclusions from the bottom-up analysis with TargetScan dataset, with the upper cut-off of the target gene size (number) decreasing, the significance of PPI enrichment of gene pairs involved in crosstalk motifs increased. Blue: single-regulation; Green: co-regulation; Red: crosstalk; Light blue: independent.

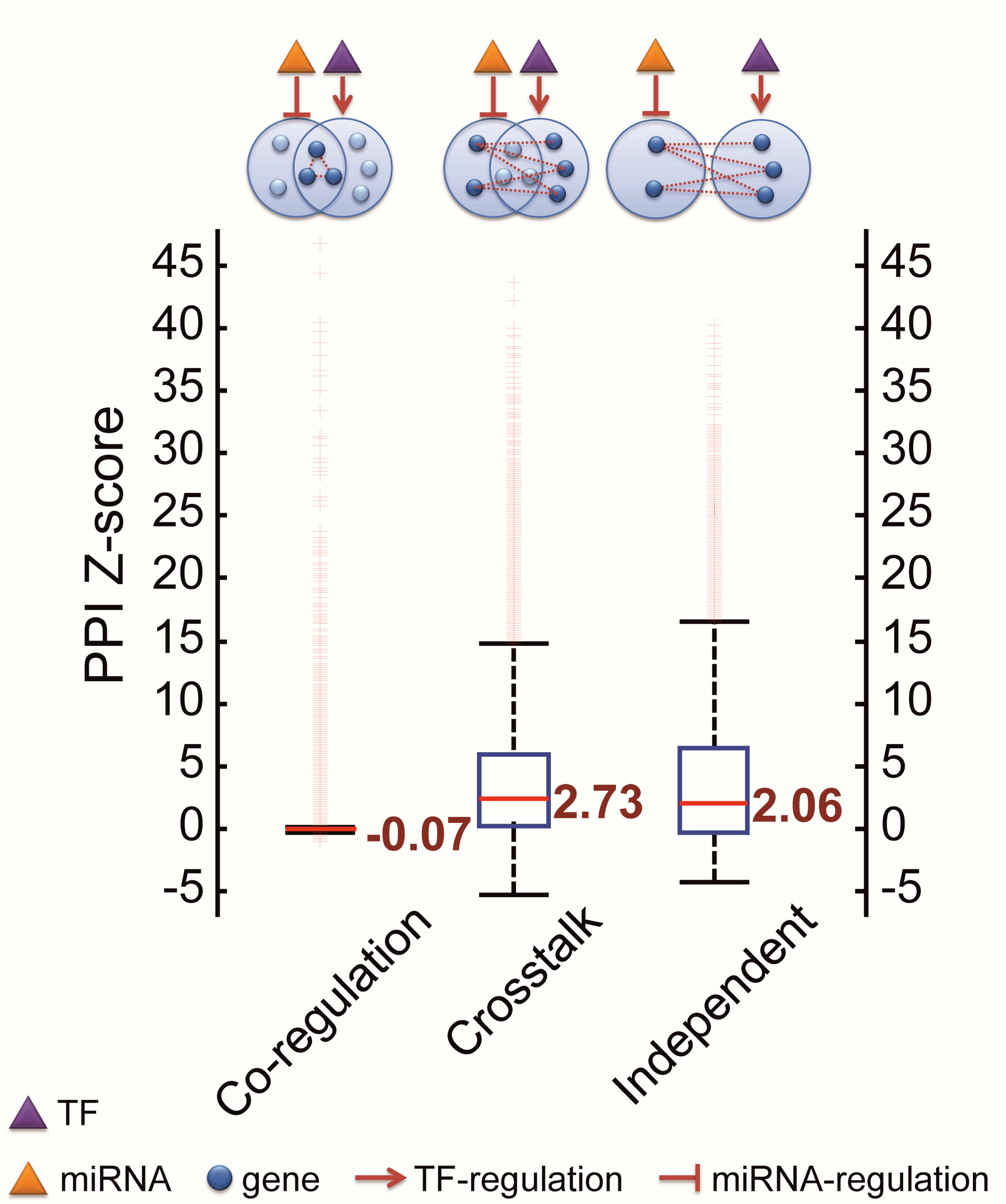

Figure S11. PPI enrichment analysis for 3 types of motifs of miRNA-TF pairs with miRBase and the union of TRED and UCSC dataset and HPRD PIN. Consistent with the conclusions from the top-down analysis with TargetScan dataset, crosstalk motif displayed the most significant correlation with PPI.

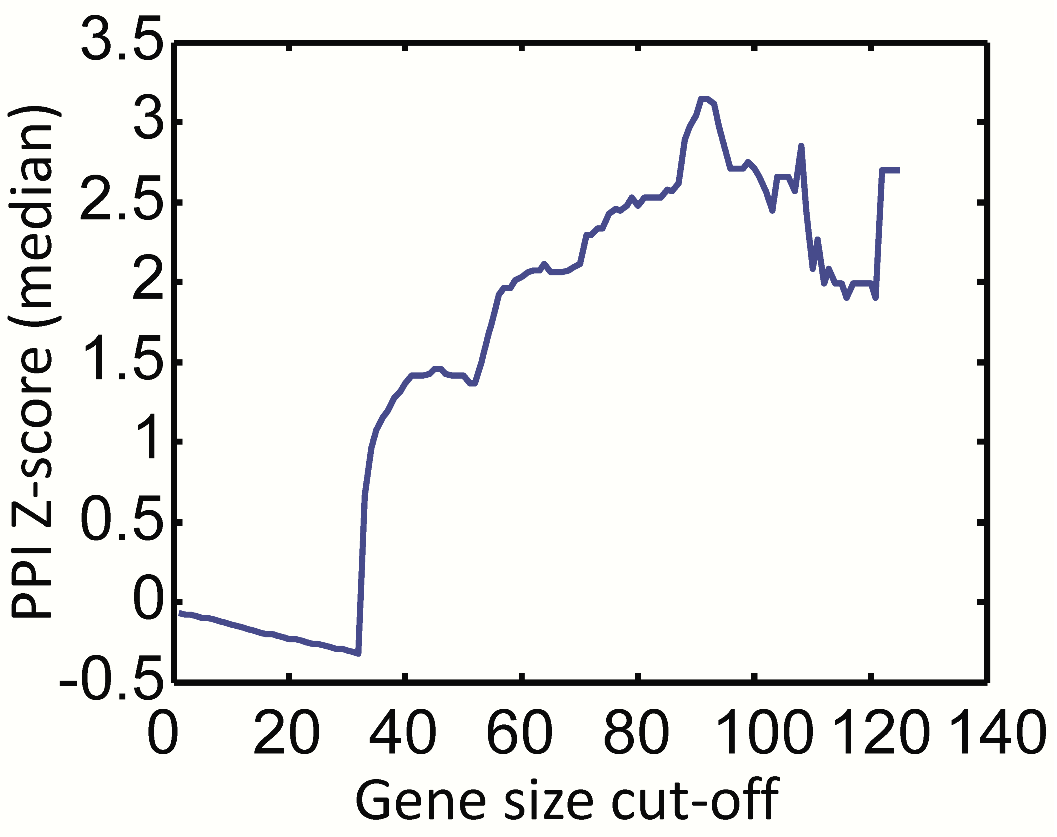

Figure S12. Co-target size filtration for co-regulation motifs of miRNA-TF pairs with miRBase and the union of TRED and UCSC dataset and HPRD PIN.

Consistent with the conclusions from the top-down analysis with TargetScan dataset, the z-score of co-regulation motif displayed a positive correlation with the cut-off of the co-target gene size (number). In other words, with the lower cut-off of the co-target gene size (number) increasing, the significance of PPI enrichment of co-regulation motifs also increased.

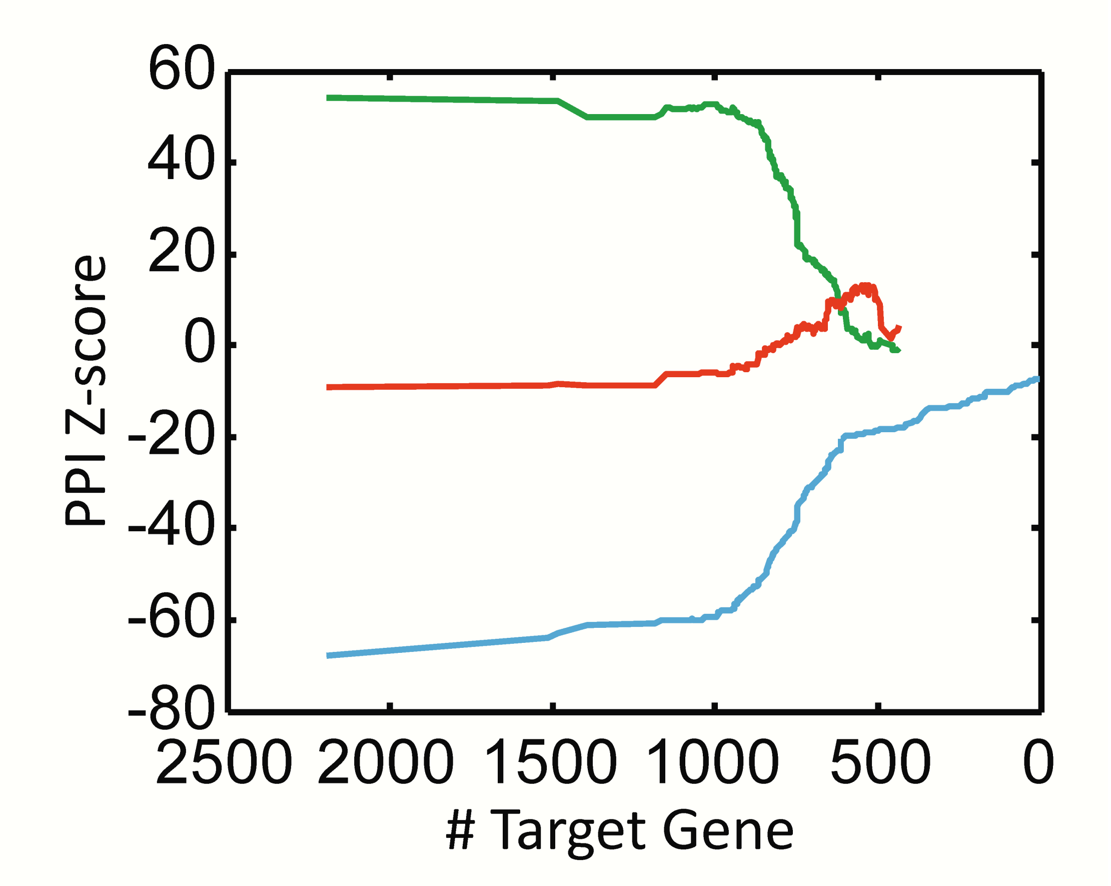

Figure S13. The correlation between the number of target genes and the z-score of PPI enrichment by using miRBase and the union of TRED and UCSC dataset and HPRD PIN.

Consistent with the conclusions from the bottom-up analysis with TargetScan dataset, with the upper cut-off of target gene size (number) decreasing, the significance of PPI enrichment of gene pairs involved in crosstalk motifs increased. The decreasing trend of PPI z-score in the tail might be due to the small sample size. Green: co-regulation; Red: crosstalk; Light blue: independent.

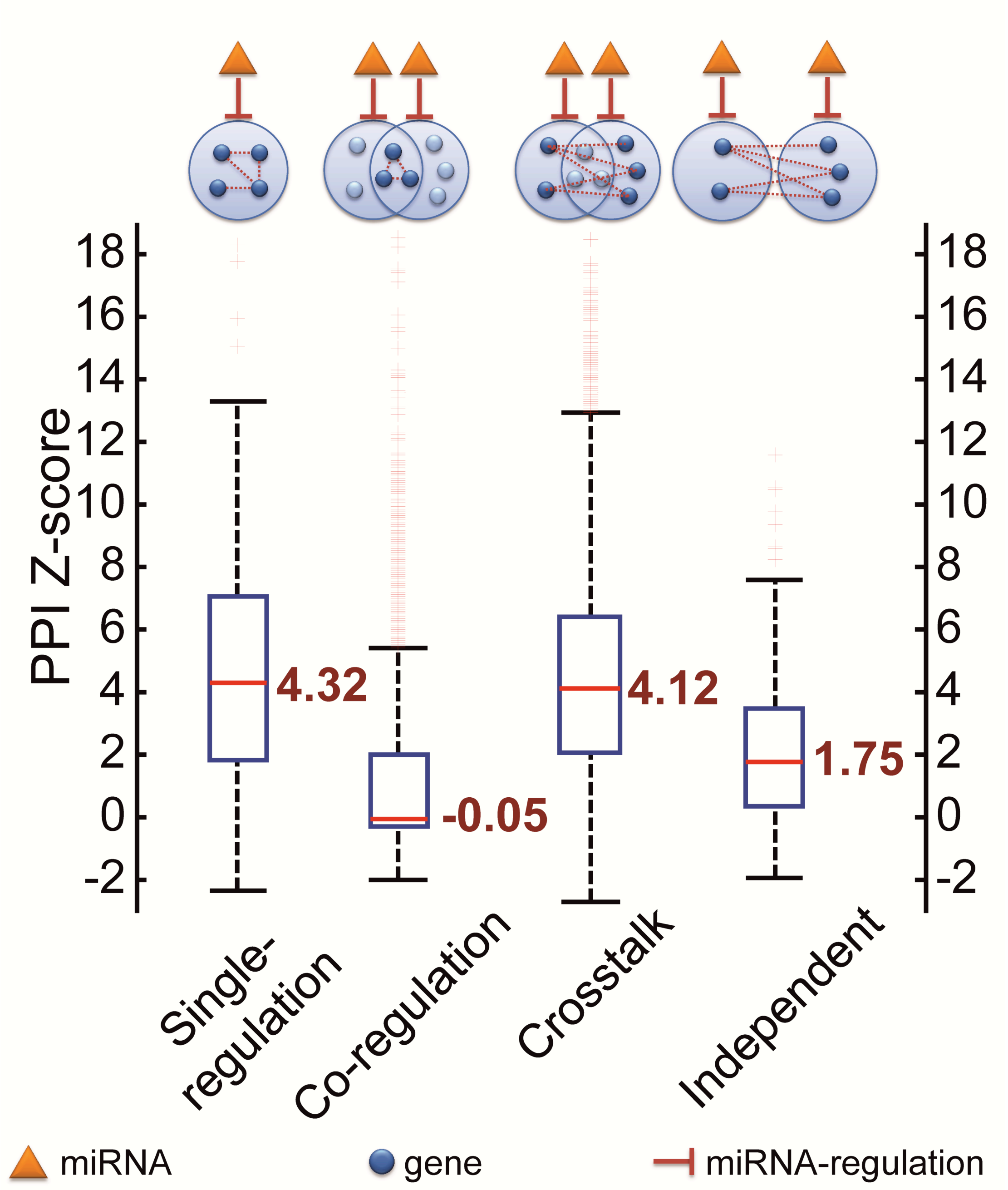

Figure S14. PPI enrichment analysis for 4 types of motifs of miRNA-miRNA pairs with TargetScan dataset and the union PIN of HPRD and BioGRID dataset.

This conclusion is consistent with the results from the top-down analysis with HPRD PIN.

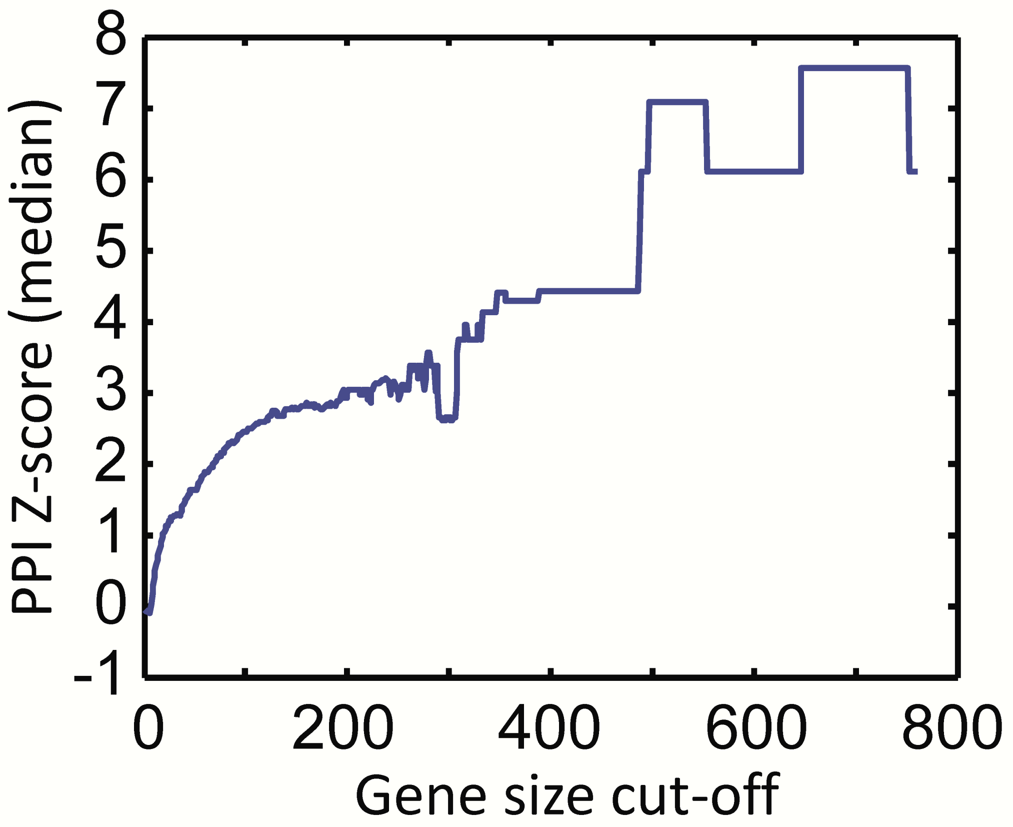

Figure S15. Co-target size filtration for co-regulation motifs of miRNA-miRNA pairs with TargetScan dataset and the union PIN of HPRD and BioGRID. Consistent with the conclusions from the top-down analysis with HPRD PIN, the z-score of co-regulation motif displayed a positive correlation with the cut-off of the target gene size (number). In other words, with the lower cut-off of the co-target gene size (number) increasing, the significance of PPI enrichment of co-regulation motifs also increased.

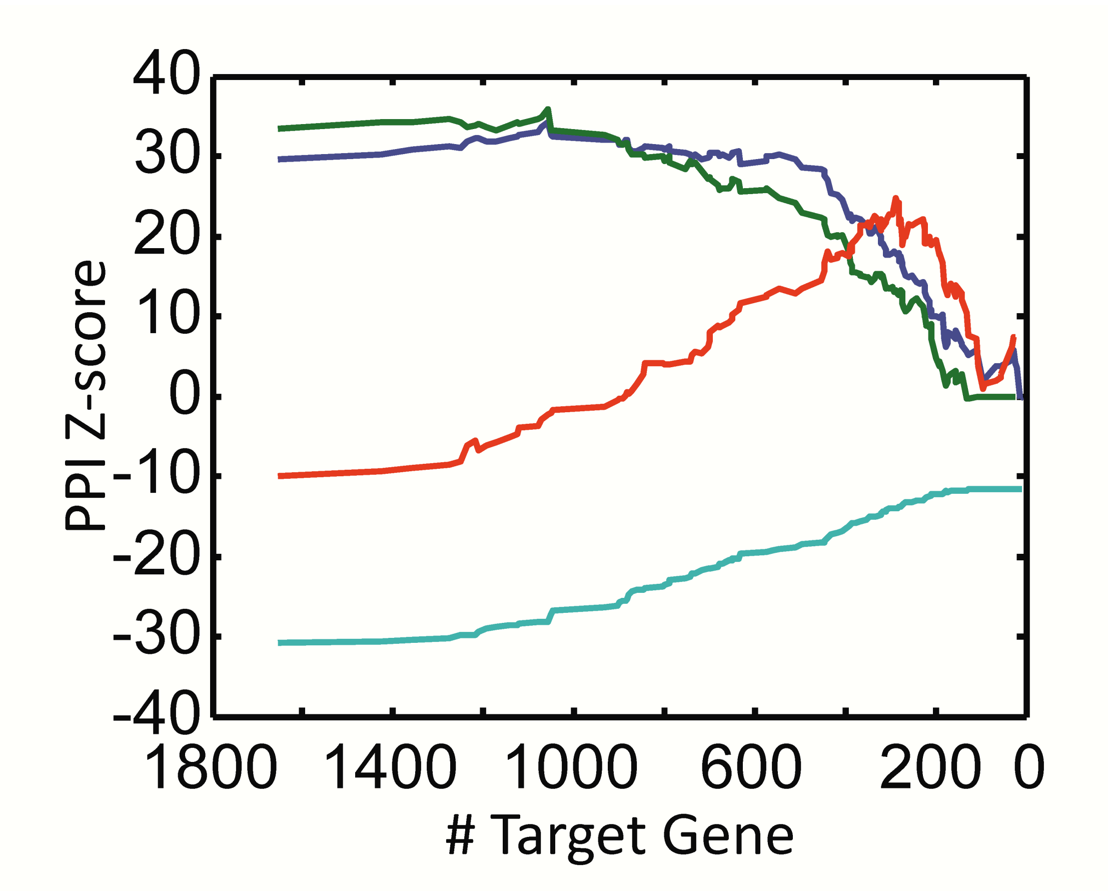

Figure S16. The correlation between the number of target genes and the z-score of PPI enrichment by using TargetScan dataset and the union PIN of HPRD and BioGRID database.

Consistent with the conclusions from the bottom-up analysis with HPRD PIN, with the upper cut-off of the target gene size (number) decreasing, the significance of PPI enrichment of gene pairs involved in crosstalk motifs increased. The decreasing trend of PPI z-score in the tail might be due to the small sample size. Blue: single-regulation; Green: co-regulation; Red: crosstalk; Light blue: independent.

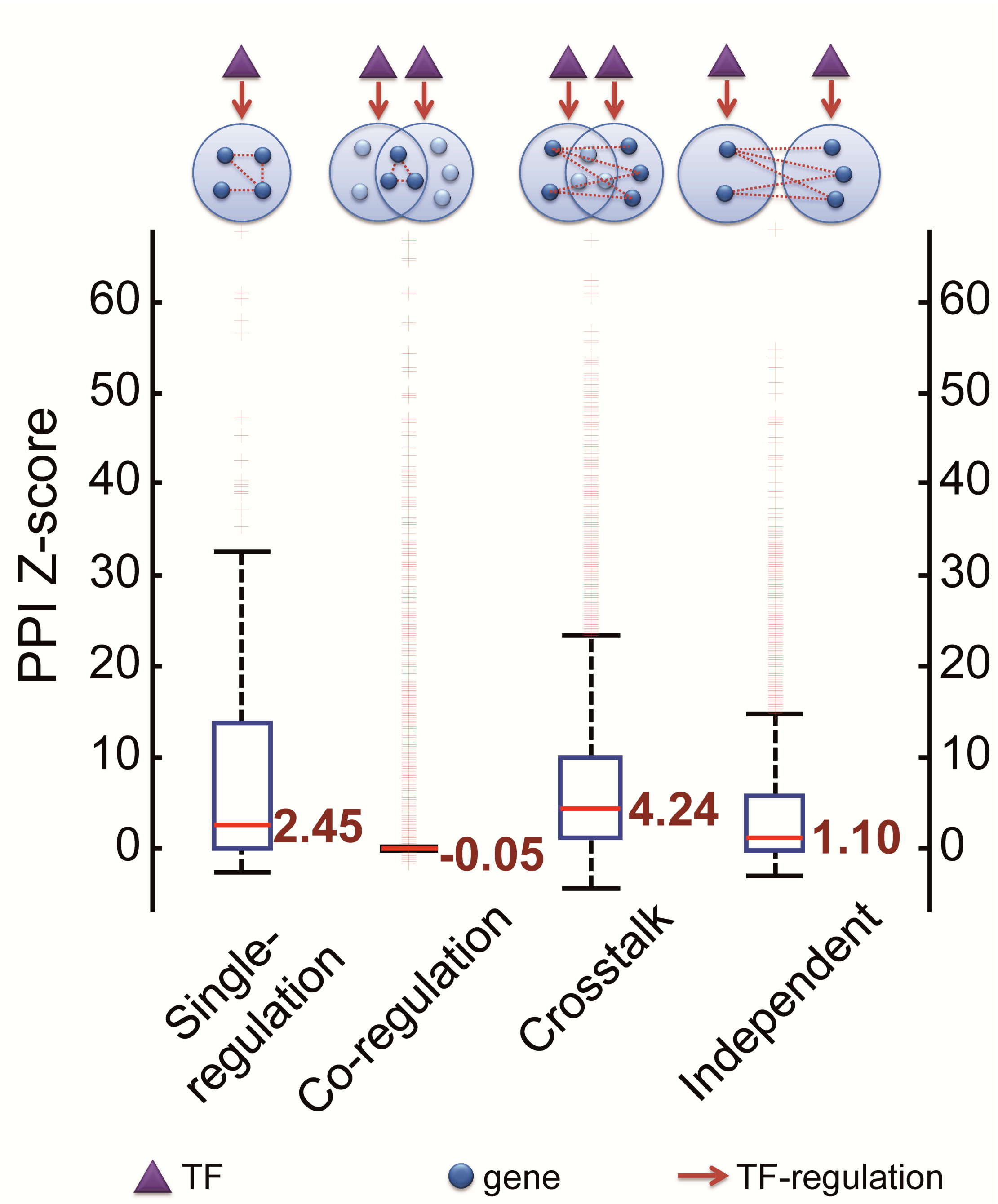

Figure S17. PPI enrichment analysis for 4 types of motifs of TF-TF pairs with the union of UCSC and TRED datasets and the union PIN of HPRD and BioGRID dataset.

The crosstalk motifs displayed a consistent conclusion with the top-down analysis by using HPRD PIN.

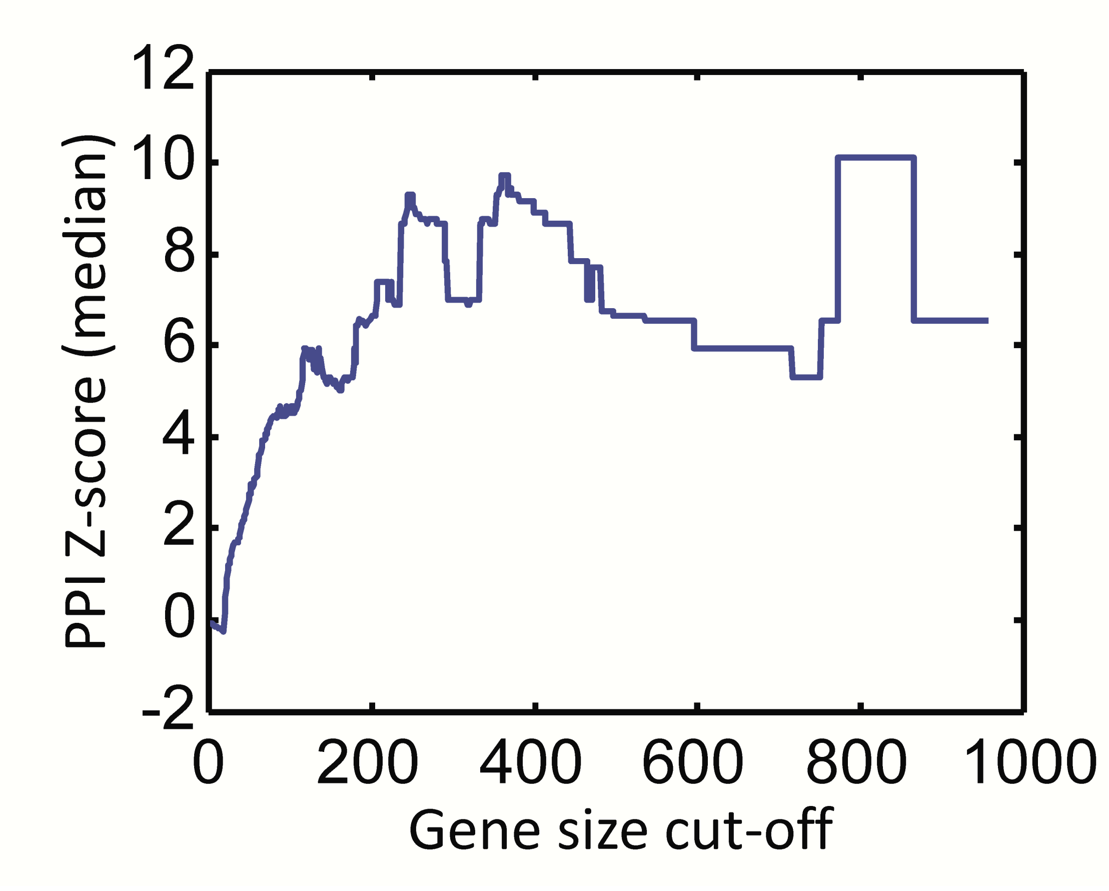

Figure S18. Co-target size filtration for co-regulation motifs of TF-TF pairs with the union of UCSC and TRED datasets and the union PIN of HPRD and BioGRID.

Consistent with the conclusions from the top-down analysis with HPRD PIN, the z-score of co-regulation motif displayed a positive correlation with the cut-off of the target gene size. When the cut-off of the gene size went larger, the tested samples were getting more and more similar with population. Therefore, the PPI z-score decreased in the tail. In other words, with the lower cut-off of the co-target gene size (number) increasing, the significance of PPI enrichment of co-regulation motifs also increased.

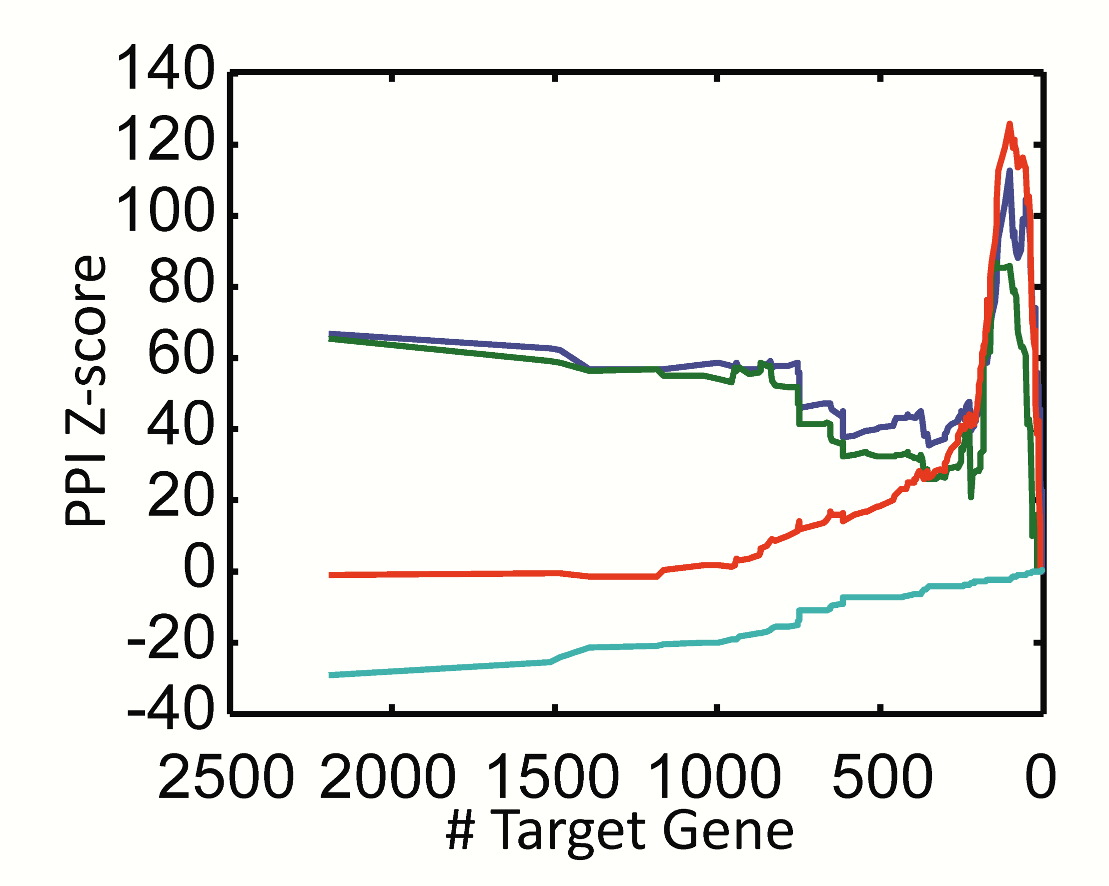

Figure S19. The correlation between the number of target genes and the z-score of PPI enrichment by using the union of UCSC and TRED datasets and the union PIN of HPRD and BioGRID database.

Consistent with the conclusions from the bottom-up analysis with HPRD PIN, with the size of target gene set decreasing, the significance of PPI enrichment of gene pairs involved in crosstalk motifs increased. The decreasing trend of PPI z-score in the tail might be due to the small sample size. Blue: single-regulation; Green: co-regulation; Red: crosstalk; Light blue: independent.

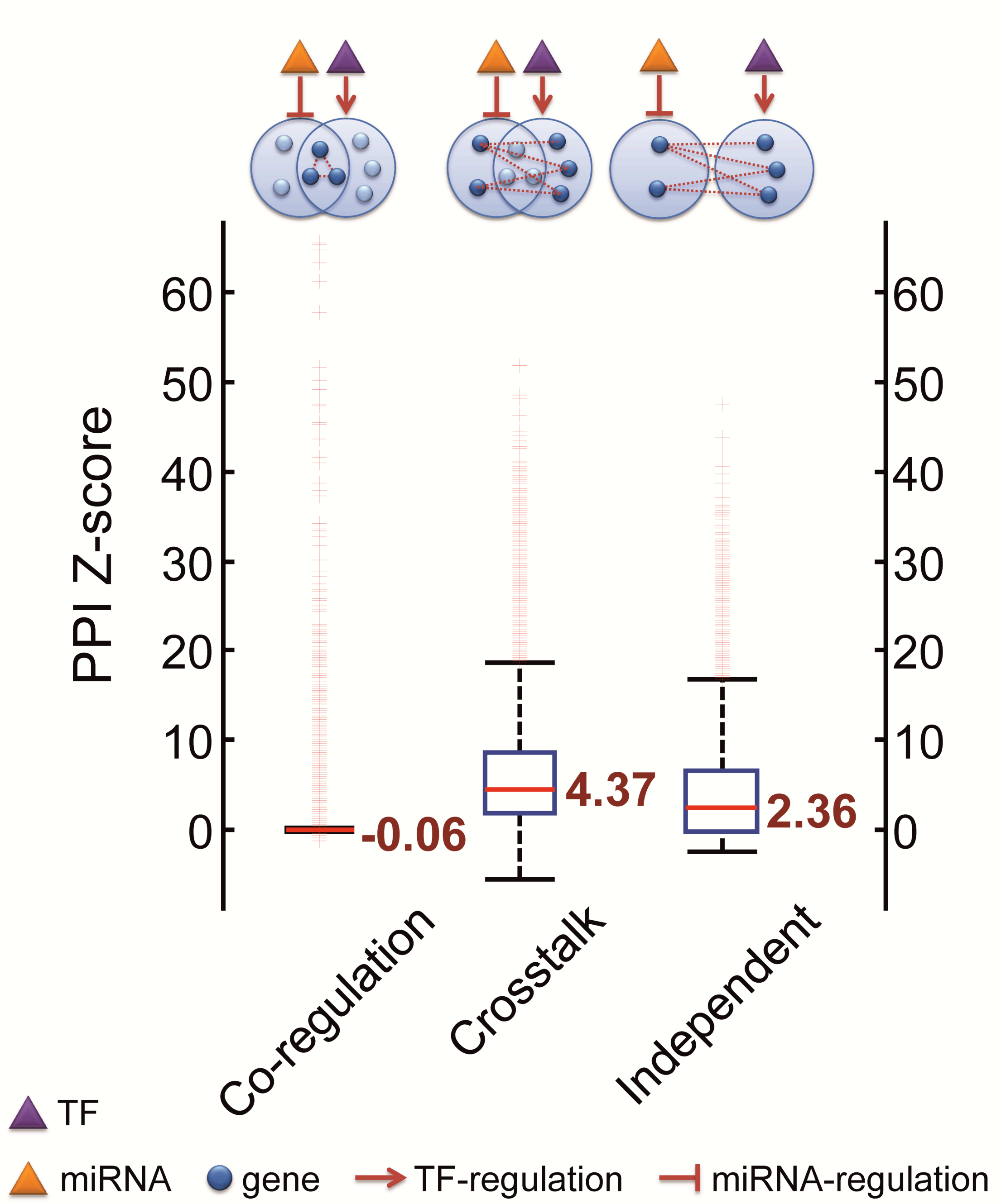

Figure S20. PPI enrichment analysis for 3 types of motifs of TF-miRNA pairs with TRED and TargetScan dataset and the union PIN of HPRD and BioGRID dataset.

Consistent with the conclusions from the top-down analysis with the HPRD PIN, crosstalk motif displayed the most significant correlation with PPI.

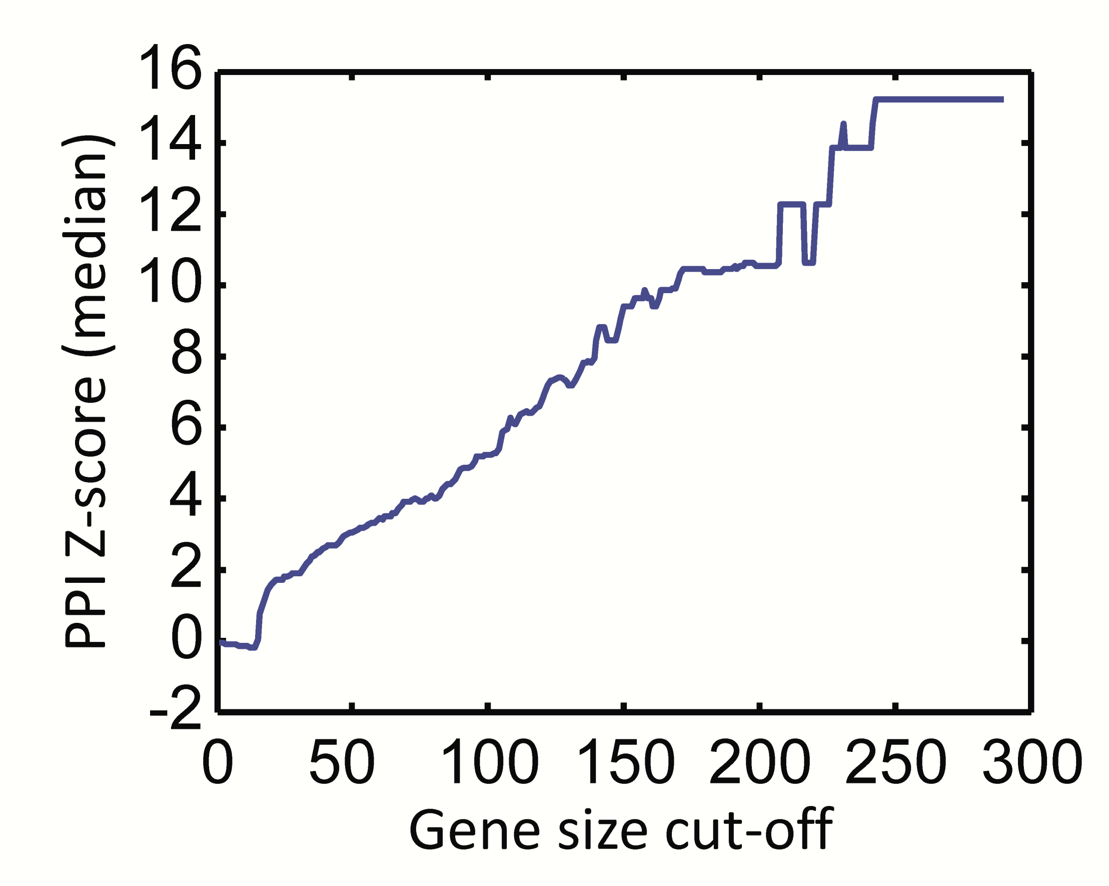

Figure S21. co-target size filtration for co-regulation motifs of TF-miRNA pairs with TRED and TargetScan dataset and the union PIN of HPRD and BioGRID database.

Consistent with the conclusions from the top-down analysis with the HPRD PIN, the z-score of co-regulation motif displayed a positive correlation with the cut-off of the co-target gene size (number). When the cut-off of the gene size went larger, the tested samples were getting more and more similar with population. Therefore, the PPI z-score decreased in the tail. In other words, with the lower cut-off of co-target gene size (number) increasing, the significance of PPI enrichment of co-regulation motifs also increased.

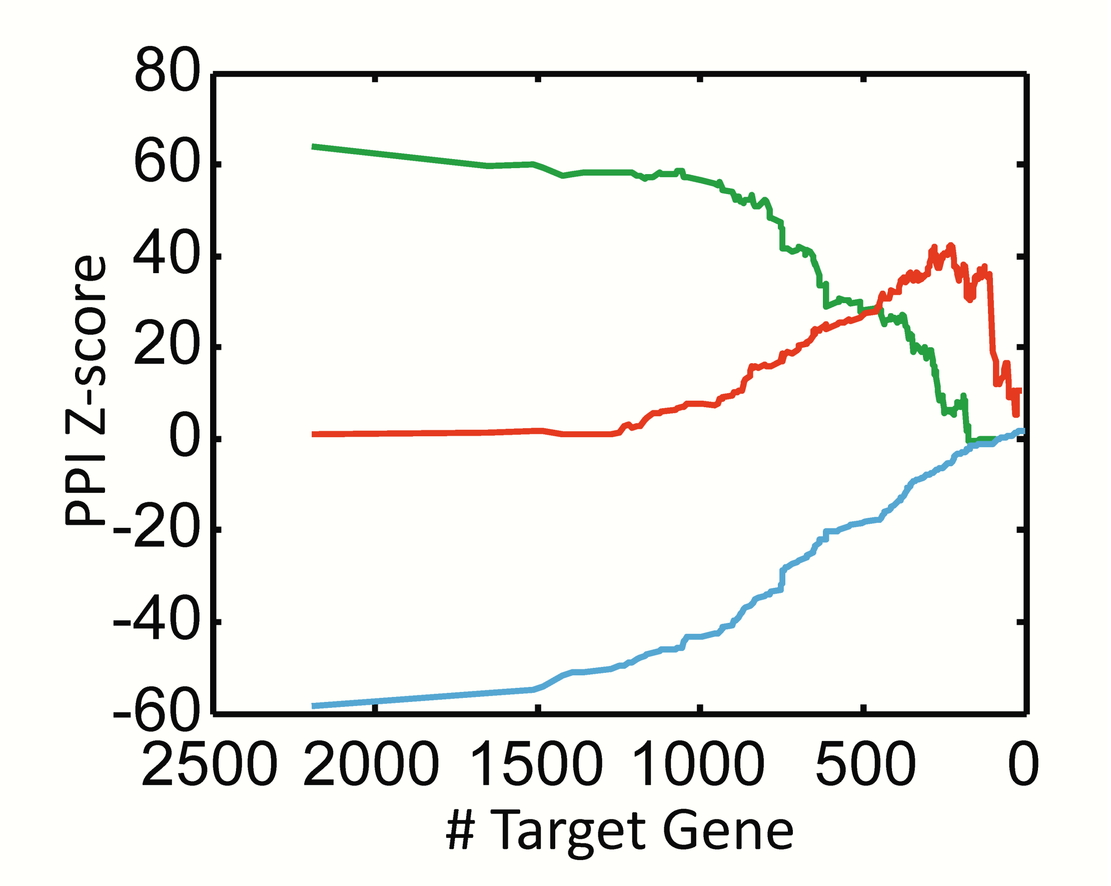

Figure S22. The correlation between the number of target genes and the z-score of PPI enrichment by using the union of UCSC and TRED and TargetScan dataset and the union PIN of HPRD and BioGRID database.

Consistent with the conclusions from the bottom-up analysis with HPRD PIN, with the upper cut-off the target gene size (number) decreasing, the significance of PPI enrichment of gene pairs involved in crosstalk motifs increased. The decreasing trend of PPI z-score in the tail might be due to the small sample size. Green: co-regulation; Red: crosstalk; Light blue: independent.

Tables

Table S1. Filtered PPI z-score and coverage of gene pairs involved in 4 types of motifs with miRBase database and HPRD PIN.

Motifs	Single-regulation	Co-regulation	Crosstalk	Independent
PPI z-score	19.30	30.00	-0.14	-17.70
Filtered z-score	8.19	5.02	7.15	-1.97
Coverage	45.60%	23.70%	99.80%	54.30%
Filtered coverage	5.49%	0.53%	51.40%	94.50%

*Motifs with miRNA-miRNA pairs

*The upper limit of target gene size for filtration is 642

Table S2. Filtered PPI z-score and coverage of gene pairs involved in 3 types of motifs with miRBase and the union of UCSC and TRED datasets and HPRD PIN.

Motifs	Co-regulation	Crosstalk	Independent
PPI z-score	54.32	-9.08	-67.85
Filtered z-score	29.03	3.98	-38.31
Coverage	8.87%	99.03%	57.20%
Filtered coverage	1.20%	75.22%	83.34%

*Motifs with TF-miRNA pairs

* The upper limit of target gene size for filtration is 750

Table S3. Filtered PPI z-score and coverage of gene pairs involved in 4 types of motifs with TargetScan database and the union PIN of HPRD and BioGRID database.

Motifs	Single-regulation	Co-regulation	Crosstalk	Independent
PPI z-score	29.59	33.49	-10.06	-30.83
Filtered z-score	30.37	28.48	4.34	-22.63
Coverage	25.68%	9.14%	96.60%	74.32%
Filtered coverage	10.48%	2.04%	68.44%	89.52%

*Motifs with miRNA-miRNA pairs

* The upper limit of target gene size for filtration is 752

Table S4. Filtered PPI z-score and coverage of gene pairs involved in 4 types of motifs with the union of UCSC and TRED datasets and the union PIN of HPRD and BioGRID database.

Motifs	Single-regulation	Co-regulation	Crosstalk	Independent
PPI z-score	66.57	65.14	-1.35	-29.26
Filtered z-score	56.53	55.86	4.35	-17.69
Coverage	16.19%	4.31%	96.65%	83.81%
Filtered coverage	8.91%	1.34%	79.80%	91.09%

*Motifs with TF-TF pairs

* The upper limit of target gene size for filtration is 870

Table S5. Filtered PPI z-score and coverage of gene pairs involved in 4 types of motifs with the union of UCSC and TRED datasets and TargetScan database and the union PIN of HPRD and BioGRID database.

Motifs	Co-regulation	Crosstalk	Independent
PPI z-score	63.89	1.01	-58.49
Filtered z-score	52.04	10.54	-38.00
Coverage	6.65%	97.42%	73.52%
Filtered coverage	2.04%	78.38%	85.26%

*Motifs with TF-miRNA pairs

* The upper limit of target gene size for filtration is 870

Methods

Statistical analysis of significance scores for regulatory motifs

To calculate the significance scores of protein-protein interaction (PPI) enrichment, we utilized the HPRD [1] protein interaction network (PIN) as population. Therefore, the expected probability of PPI can be defined as:

$$\mu = p = \frac{PPI_{all}}{N}, 0 \leqq \mu \leqq 1$$

where μ and p denotes the expected probability of PPI in HPRD PIN, PPI_{all} represents the number of PPI and N represents the number of gene pairs in HPRD PIN. Then, Bernoulli distribution was applied to estimate the standard deviation (σ) of this population.

$$\sigma = \sqrt{p(1-p)}, 0 \leq \sigma \leq 0.5$$

In other words, we suppose PPI gain and lost followed the binomial distribution. Therefore, the null hypothesis, H_0, can be established.

$$H_0 : \mu_0 = \mu$$

The statistical meaning of this null hypothesis is that the probability of each gene pairs which gains a PPI in HPRD PIN based on a random process is μ. Next, regarding the interested gene set, the expected probability of PPIs can be defined as:

$$x_1, x_2, x_3, \dots, x_n \text{ , } x_i = \begin{cases} 0 & \text{, without PPI} \\ 1 & \text{,with PPI} \end{cases}$$

$$\bar{X} = \frac{1}{n}\sum_{i=1}^{n} x_i, 0 \leq \bar{X} \leq 1$$

where $\bar{X}$ denotes the expected probability of PPIs of the interested gene set; x_i represents whether gene pair i forms a PPI; n represents the number of gene pairs among the interested gene set. Finally, the significance score can be calculated from the Z-normalization. The z-score is defined as:

$$z_{score} = \frac{\bar{X} - \mu}{\sigma / \sqrt{n}}$$

Larger $|z_{score}|$ represents that interested gene set is with higher probability of rejecting the null hypothesis. In other words, the interested gene set with larger $|z_{score}|$ means more significant correlation with PPI.

Investigating the functional roles of crosstalk motifs

The functions of TF were assigned by annotated Gene Ontology (GO) [2] terms of the corresponding TF encoding gene. The functional roles of miRNA in PIN were defined as significantly over-represented GO biological processes of its target genes. Hypergeometric test was used to determine which GO terms were significantly over-represented ($P \leq 0.001$). The hypergeometric probability distribution is described as:

$$P(X = k) = \frac{\binom{m}{k}\binom{N-m}{n-k}}{\binom{N}{n}}$$

where X denotes the evaluated functional category in GO; N represents the number of GO annotated genes participated in PIN; n represents the number of genes which are annotated as the evaluated GO functional category in PIN; m represents the number of GO annotated genes participated in tested group of genes. Thus, this formula calculates the probability of the evaluated functional category covering k genes in that network. The calculated P-value was then adjusted by applying the Benjamini and Hochberg multiple testing procedures to control the false discovery rate (FDR) [3]. In order to obtain more specific functions of regulators, only those GO terms with GO level greater than or equal to 6 were considered to be potential biological functions of regulators. The functional similarity was defined as the ratio of common enriched functions between two regulators.

To investigate the underlying biological processes of crosstalk motifs, we applied the functional enrichment analysis to identify the associated functions of PPIs between two private targets. Shared GO biological processes between a pair of genes that encode a pair of interacting proteins were considered as the associated functions of the corresponding PPI. The significance of the associated function of PPI was determined by p-value (≤ 0.001) derived from a modified hypergeometric test:

$$P_e(X = k_e) = \frac{\binom{m_e}{k_e}\binom{N_e - m_e}{n_e - k_e}}{\binom{N_e}{n_e}}$$

where *e* is the abbreviation of PPIs with associated functions. Each symbol represents the same meaning with the previous one in the original hypergeometric probability distribution, but the counting targets are changed from genes to PPIs. The calculated *P*-value was also adjusted by applying the Benjamini and Hochberg multiple testing procedures to control the FDR. In order to obtain more specific functions of PPIs, only those GO terms with GO level greater than or equal to 6 were considered to be associated biological functions of PPIs. The underlying biological processes of crosstalk motifs were referred to corresponding significantly enriched associated functions of PPIs between two private target sets.

Network properties

A PIN can be represented as an undirected graph G(V,E) that consists of a set of nodes V and a set of edges E. Each node $u \in$ V represents a unique protein encoded by a gene *u*, while each edge (u, v) $\in$ E represents an observed PPI between two proteins *u* and *v*. In this study, five network properties in HPRD PIN were analyzed: degree, closeness centrality, density, path length, and clique level [4]. The degree is the number of the observed PPIs of a given protein in HPRD PIN. The density of a

network is the ratio of the observed PPIs to the maximal connections among nodes, and denser network possessed higher density. The path length between two nodes in a network is the number of edges in a shortest path connecting them. In addition, the characteristic path length of a network is the average path length between any two nodes in the observed network. A clique is a complete network in which each pair of nodes is connected by an edge. If a clique is not a subgraph of any other clique, then this clique is called a maximal clique. The clique level of a node is defined as the largest size of maximal clique that the node can join. Closeness is the sum of inverse shortest paths from a specific node to other nodes.

References

1. Goel R, Muthusamy B, Pandey A, Prasad TS: **Human protein reference database and human proteinpedia as discovery resources for molecular biotechnology.** *Mol Biotechnol* 2011, **48:**87-95.
2. Ashburner M, Ball CA, Blake JA, Botstein D, Butler H, Cherry JM, Davis AP, Dolinski K, Dwight SS, Eppig JT, et al: **Gene ontology: tool for the unification of biology. The Gene Ontology Consortium.** *Nat Genet* 2000, **25:**25-29.
3. Benjamini Y, Yekutieli D: **The control of the false discovery rate in multiple testing under dependency.** *Ann Stat* 2001, **29:**1165-1188.
4. Wasserman S, Faust K: **Social Network Analysis: Methods and Applications.** *Cambridge University Press* 1994.
